# Singlet exciton fission in a modified acene with improved stability and high photoluminescence yield

**DOI:** 10.1038/s41467-021-21719-x

**Published:** 2021-03-09

**Authors:** Peter J. Budden, Leah R. Weiss, Matthias Müller, Naitik A. Panjwani, Simon Dowland, Jesse R. Allardice, Michael Ganschow, Jan Freudenberg, Jan Behrends, Uwe H. F. Bunz, Richard H. Friend

**Affiliations:** 1 Cavendish Laboratory, University of Cambridge, JJ Thompson Avenue, Cambridge, UK; 2 Organisch-Chemisches Institut, Ruprecht-Karls-Universität Heidelberg, Im Neuenheimer Feld 270, Heidelberg, Germany; 3 Berlin Joint EPR Lab, Fachbereich Physik, Freie Universität Berlin, Berlin, Germany; 4 Present Address: Pritzker School of Molecular Engineering, University of Chicago, Chicago, IL USA

**Keywords:** Molecular electronics, Solar cells, Magnetic properties and materials

## Abstract

We report a fully efficient singlet exciton fission material with high ambient chemical stability. 10,21-*Bis(triisopropylsilylethynyl)tetrabenzo[a,c,l,n]pentacene* (TTBP) combines an acene core with triphenylene wings that protect the formal pentacene from chemical degradation. The electronic energy levels position singlet exciton fission to be endothermic, similar to tetracene despite the triphenylenes. TTBP exhibits rapid early time singlet fission with quantitative yield of triplet pairs within 100 ps followed by thermally activated separation to free triplet excitons over 65 ns. TTBP exhibits high photoluminescence quantum efficiency, close to 100% when dilute and 20% for solid films, arising from triplet-triplet annihilation. In using such a system for exciton multiplication in a solar cell, maximum thermodynamic performance requires radiative decay of the triplet population, observed here as emission from the singlet formed by recombination of triplet pairs. Combining chemical stabilisation with efficient endothermic fission provides a promising avenue towards singlet fission materials for use in photovoltaics.

## Abstract (web summary)

Designing optimised molecules for singlet fission is crucial to improve the efficiency of solar cells beyond its theoretical limit. Here, the authors investigate pentacene derivative TTBP, which exhibits high stability and luminescence yield, and find it highly suitable for exciton multiplication purposes.

## Introduction

Singlet fission, the evolution of one photoexcited singlet exciton to form a pair of triplet excitons, occurs in a subset of organic semiconductors where the energy of a singlet is approximately twice the energy of a triplet. Initially reported in 1969 in crystalline tetracene^1^, there has been a growing interest in singlet fission in recent years owing to its demonstrated ability to increase the efficiency of photovoltaic devices^2,3^. In a traditional single-junction device, all photons with energy above the bandgap will lose their excess energy to heat as the excitations relax to the band edge, before being extracted as charges. These thermalisation losses can be reduced by absorbing high-energy photons with a singlet fission chromophore and producing two triplet excitons with energy greater than or equal to the bandgap of the device (i.e., 1.1 eV for silicon). The energy of both these triplet excitons must then be transferred to the main device, either via triplet energy transfer, electron transfer, or the emission and absorption of infra-red photons^4–7^. Calculations have shown that for an optimal bandgap and singlet fission chromophore, the theoretical limit of a single-junction solar cell increases from 33% to 45% with singlet fission^8,9^. Recent experiments demonstrate that integration of tetracene with silicon enables triplet energy transfer into silicon following photon excitation in tetracene^7^, affirming the potential impact of a chemically stable alternative. The possibility of increased efficiencies from silicon tandem systems, of which singlet fission is a contender, has led to projections from the International Technology Roadmap for Photovoltaics that tandems will begin to take significant market share from single-junction c-Si modules in the next decade, with 4% in 2030^10^. One of the biggest roadblocks for realising this potential from singlet fission tandems is the stability of the singlet fission materials.

Currently, the most thoroughly studied and highest yield singlet fission systems are linear acenes and their derivatives^11–20^, but singlet fission has also been reported in indigoids^21^, rylenes^22–24^, polyenes^25–29^, diketopyrrolopyrroles^30,31^, and 1,3-diphenylisobenzofurans^32^. Tetracene and TIPS-tetracene have been of particular interest owing to the favourable triplet energy (~1.25 eV) slightly above the bandgap of silicon, and because for them fission is endothermic yet still efficient, maximising potential power output thanks to thermal energy. Here we study 10,21-bis(triisopropylsilylethynyl)tetrabenzo[*a,c,l,n*]pentacene (TTBP), a soluble acene derivative with improved ambient stability, which retains the characteristic acene behaviour of singlet fission, and also demonstrates reduced non-radiative loss pathways that would be parasitic in a solar cell. We note a recent theoretical study has shown the potential for pyrene-fused acenes for singlet fission^33^, which while not identical is a similar structural motif, and intra-molecular singlet fission has been experimentally observed in dimers of pyrene-fused azaacenes^34^, demonstrating that adding off-linear fused rings does not preclude singlet fission.

As we develop in this paper, in TTBP we find both efficient singlet fission, and also triplet fusion evident through delayed singlet emission. Thanks to the favourable energetics that make singlet fission reversible, and notably slow non-radiative decay, TTBP has a high photoluminescence quantum efficiency (PLQE) of 20%, despite singlet fission to the pair state proceeding at ~200 times the radiative rate. It follows that if used in a singlet fission tandem where triplets could be efficiently transferred to a lower bandgap solar cell (an engineering challenge yet to be solved), TTBP would have much lower non-radiative voltage losses, which limit the power conversion efficiency, than an equivalent low-PLQE, irreversible singlet fission material. This thermodynamic argument is in essence identical to the detailed balance limit for single-junction cells^9^, and we expand on this idea further later in the paper.

TTBP was synthesised in 2018 by Müller et al.^35^, who found that the two- or four-fold benzannulation of heptacene, hexacene, and pentacene improved their stability. The addition of the branched triphenylene “butterfly-wings” extends the π-conjugated system, but not as significantly as a lone linear benzene annulation would do, causing TTBP to have similar energy levels to tetracene (Supplementary Fig. 1). There is also a similarity to the energetics of rubrene, another acene derivative capable of both singlet fission and triplet fusion, although unlike TTBP, rubrene is highly unstable, and has not been reported to show the same high photoluminescence yield in the solid state, but can undergo highly efficient triplet-triplet annihilation (TTA) on single rubrene molecules when in a dilute form^36^. Through a combination of optical and spin-resonance experiments, we show that singlet fission occurs quantitatively in solid samples that also have high photoluminescence efficiency. We propose that, as in a typical single-junction photovoltaic, a high photoluminescence efficiency is key to reducing voltage losses due to non-radiative decay in a potential singlet fission solar cell.

## Results and discussion

**Fig. 1:**
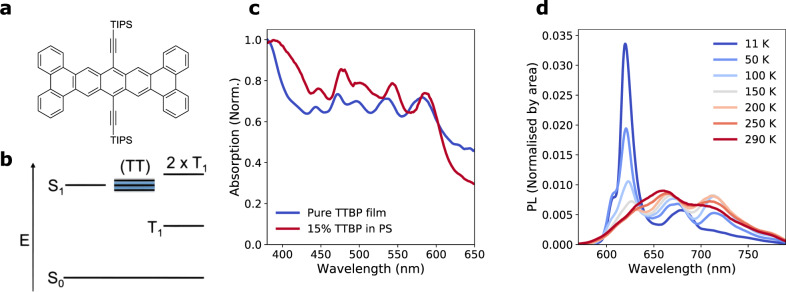
Molecular structure and stead-state characterisation of TTBP. **a** Molecular structure of TIPS-tetrabenzo[a,c,l,n]pentacene (TTBP). **b** Schematic energy level diagram for endothermic singlet fission in TTBP. **c** Absorption spectrum of a pure TTBP film (blue) and a 15% blend of TTBP in polystyrene (red) at room temperature. The measured absorption at wavelengths longer than ~600 nm is due to scattering and reflectance. **d** Temperature-dependent time-integrated photoluminescence, using a pulsed excitation source at 500 nm, pulse duration 200 fs, 1 kHz repetition, from 11 K to room temperature. Colours blue to red indicate temperature, from lowest (11 K) to highest (290 K).

The molecular structure of TTBP is given in Fig. 1a, with an energy level diagram showing ordering of singlet and triplet energy levels, where singlet fission to a pair of uncorrelated triplets (2 × T_1_) is overall endothermic, and mediated by a pair-state manifold (TT state) that is approximately isoenergetic with the singlet (Fig. 1b). Figure 1c shows continuous-wave (CW) optical absorption of 100% TTBP films and 15% TTBP in a polystyrene host. Both show some scattering, evident in the reduced transmission at long wavelengths. Solid-state films show a 25 nm red-shift in the absorption onset relative to the solution (Supplementary Fig. 1), indicating significant intermolecular interaction in the solid state. In this work, we use either 100% TTBP films or TTBP/polystyrene (PS) blend films, which exhibit comparable optical properties (see Fig. 1c), in order to reduce scattering. The level of aggregation in these films can be inferred from their photoluminescence spectra, as shown in Supplementary Figs. 3 and 4. The strong majority of TTBP is aggregated at 5% concentration in PS, with a minority of dispersed molecules, meaning that the transient absorption (TA) experiments for which the polystyrene blends were designed are dominated by the signal from aggregated TTBP.

Figure 1d shows the temperature dependence of the photoluminescence; we discuss the strong temperature dependence later, and note here that the sharper and stronger low-temperature emission is similar to that of tetracene^14^.

**Fig. 2:**
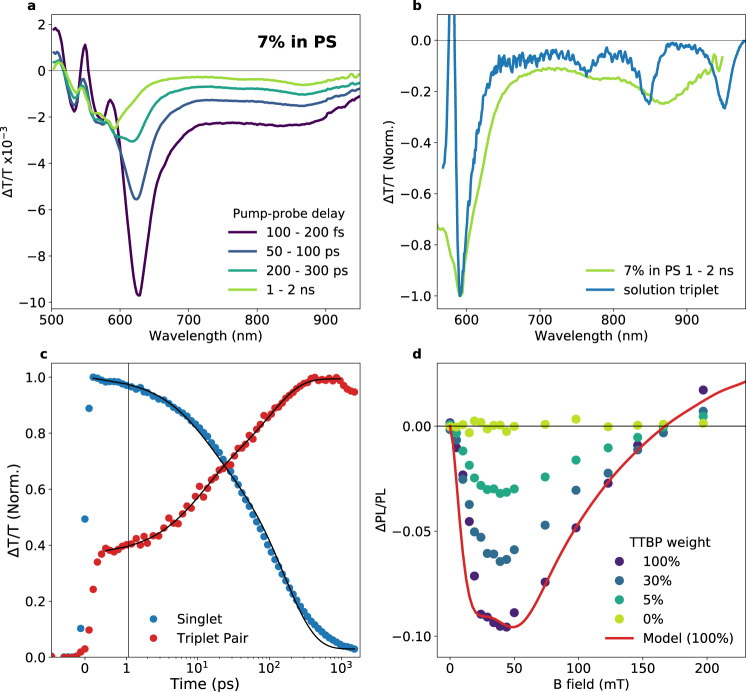
Triplet pair formation in TTBP. **a** Time-cuts from ultrafast, room temperature transient absorption with a 250 fs, 38 kHz, 500 nm pump for a 7% TTBP film. **b** Comparison of the 1–2 ns time-cut with the solution triplet spectrum (using *N*-methylfulleropyrrolidine as sensitiser) showing a red-shift and broadening in the near IR absorptions. **c** Kinetics of the singlet (blue) and triplet (red) spectra, separated using a Genetic Algorithm (see Methods). Solid lines are biexponential fits, see text. **d** Magnetic field effect on room temperature steady-state photoluminescence using 520 nm CW excitation. Circles indicate a relative change in integrated photoluminescence intensity in a magnetic field for films of varying weight % of TTBP in polystyrene, the solid red line shows a fit for the 100% TTBP film of the Merrifield model averaged over 10,000 random angles between **B** field and molecular orientation.

TA, where samples are excited with a narrow 500 nm, 200 fs pulse, followed by a broadband probe with a controllable delay, allows the tracking of photoexcited states over time with femtosecond resolution. In TTBP/polystyrene films, the photoexcited singlet, which has a photoinduced absorption (PIA) at 620 nm, is quenched to form a triplet-like species, which shows a strong PIA at 590 nm and a series of smaller PIAs in the near IR (Fig. 2a, b). Population kinetics in Fig. 2c are extracted from the TA data shown in Fig. 2a using a Genetic Algorithm (see Supplementary Fig. 11 for species associated spectra). Triplet pair formation occurs on a timescale of 100 ps or faster; the solid lines in the figure show biexponential fits with time constants of 11 ps and 160 ps for the singlet decay, and 10 ps and 100 ps for the triplet rise. We note that the overall singlet population decay may be slower than the singlet fission rate owing to a sub-population of isolated molecules in the polystyrene blend that does not undergo singlet fission and hence is expected to decay with a 22 ns lifetime (Supplementary Fig. 14). The apparent initial rise of the triplet pair signal to 0.4 within the instrument response function of the experiment could be coherent pair formation, as seen by Stern et al. for TIPS-tetracene^19^.

Using the relative absorption cross-sections of the PIAs at 620 nm for singlets and 590 nm for triplets, we calculate the initial fission process to be close to full efficiency at low fluences where singlet-singlet annihilation is minimised (details in Supplementary Note 4). This is expected, given that the radiative lifetime from a dilute (0.3%) film in PS with 97% PLQE is 22 ns (Supplementary Fig. 14), so singlet fission is approximately two orders of magnitude faster than radiative decay and there are no other competitive non-radiative pathways. The spectrum after 1 ns is a close match to the solution triplet spectrum obtained by sensitisation at 590 nm (Fig. 2b), although the NIR transitions are significantly red-shifted and broadened, a possible indication of strong electronic coupling within the triplet pair^18,19^.

The modulation of steady-state photoluminescence intensity under low magnetic field unequivocally confirms that singlet fission occurs in TTBP. A range of TTBP films, diluted to varying concentrations in polystyrene, display a clear magnetic field effect (MFE), as shown in Fig. 2d. The observed MFE exhibits the signature initial decrease in photoluminescence at low fields followed by an increase to a positive MFE, as originally observed in tetracene^1^. We note that the **B** field of minimum photoluminescence and the zero-crossing point where *PL*(**B**) = *PL*(0) are both at much higher fields than for other singlet fission materials such as tetracene^1^, rubrene^37^, anthracene^38^, and DPP^39^. Fitting to the Merrifield model described in Supplementary Note 5, we obtain the ratio *ε*, of the rate of triplet fusion (from pair-state back to singlet) to the rate of pair dissociation. The high zero-crossing point results in a fit with a high *ε* (in the case of TTBP, *ε* = 10), indicative of fast geminate triplet fusion and/or slower pair dissociation.

As shown in Supplementary Fig. 21, the MFE on PL is approximately uniform across the whole spectrum, despite the differences in kinetics in different parts of the PL spectrum (see below). This indicates that any emission from lower-energy geometries/defects also originates from excitons produced by or capable of singlet fission, and is not indicative of a singlet-trapping mechanism that is competitive with singlet fission, although it does not rule out a trap for triplet pairs. Lowering the concentration of TTBP in polystyrene reduces the magnitude of the effect, implying an increasing fraction of the total emission is from isolated molecules that do not exhibit singlet fission. However, there is a measurable MFE down to 5% by weight, indicating that even these dilute samples contain aggregates exhibiting singlet fission.

**Fig. 3:**
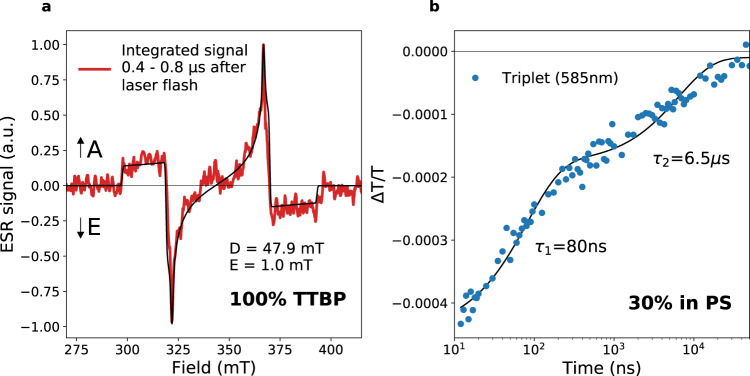
Long-lived triplets excitons in TTBP. **a** Time-resolved ESR for a dropcast film of 100% TTBP at 50 K, integrated from 0.4–0.8 µs after laser excitation at 532 nm. The sample substrate was oriented at ~45° to the **B**_**0**_ direction. The polarisation pattern of AEEAAE is consistent with singlet fission, which overpopulates the *m*_*s*_ = 0 sublevel. Black line is a simulation using EasySpin^51^ for a triplet with zero-field splitting parameters *D* = 47.9 mT, *E* = 1.0 mT, and *g* = 2.000, linewidth = 0.4 mT. **b** Triplet decay kinetics in a 30% TTBP in polystyrene film, from room temperature ns-resolution transient absorption using a 1 ns, 500 Hz, 532 nm pump in the visible region. An approximately biexponential decay is observed with time constants of 83 ns and 6.5 µs, likely from geminate and non-geminate triplet decay.

Electron spin resonance (ESR) uses microwaves to probe resonances of transitions between the sublevels of non-zero spin states such as doublets (*S* = 1/2), triplets (*S* = 1), and quintets (*S* = 2). Time-resolved ESR (trESR) couples this with a pulsed laser to observe the ESR signatures of photoexcited states, and their lifetimes. The relative sublevel populations (e.g., for a triplet state, the populations with spin projection quantum number along the magnetic field direction *m*_*s*_ = ±1,0) of a state dictate the polarisation pattern of the ESR signal. This pattern of microwave emission and absorption across magnetic field, therefore, offers insights into its formation mechanism. In TTBP, we observe triplets in trESR with a polarisation pattern AEEAAE (Fig. 3a), indicating a relative overpopulation of the *m*_*s*_ = 0 triplet sublevel relative to +1 or −1^21^, a further unambiguous confirmation that the triplets are formed by singlet exciton fission to an overall spin-singlet pair-state, which subsequently dissociates (triplets formed by intersystem crossing instead of singlet fission, as in dilute TTBP solution, would have an EEEAAA polarisation pattern, see Supplementary Fig. 29). In other acene derivatives where a triplet pair-state is formed, trESR has been used to observe triplet pairs in spin-quintet states^40–42^. The absence of quintets in TTBP from 11–100 K suggests that either the triplet pairs in TTBP dissociate or decay faster than the time resolution of trESR (~300 ns), and thus we only observe dissociated triplets, or *J* is very large, preventing mixing to the quintet manifold. The monomolecular decay of photoluminescence on a ~65 ns timescale suggests that the pair-state is too short lived to be observed in trESR. The intensity of the ESR signal decreases by a factor of 2 from 100 K to 11 K (Supplementary Fig. 22), despite the fact that lower temperatures increase the Q-factor of the resonator and extend the spin relaxation times, which should both increase the trESR signal intensity. We attribute this to the fact that at lower temperatures, there is less thermal energy to separate the triplet pair-state into free triplets. This interpretation is also consistent with the substantial increase in PL yield at lower temperatures (Supplementary Fig. 10).

The trESR spectrum shows an orientation dependency with the rotation of the substrate relative to the external magnetic field (**B**_**0**_) direction, indicating orientationally selective crystallisation of molecules relative to the substrate (Supplementary Fig. 23). Although we do not rule out a contribution to the rotational dependence from the presence of both of the two known polymorphs of TTBP, reported by Müller et al.^35^, we were able to simulate the orientation-dependent trESR spectra using a model, which represents partial ordering in our system (Supplementary Fig. 26). The simulations are consistent with the expected preferential alignment of the long axis of the conjugated core vertical to the substrate, as is typical for solution-processed small molecules that interact more strongly with each other than with the substrate. The trESR of a 10% TTBP in polystyrene sample, Supplementary Fig. 28, also shows the same orientation dependence, a further indication of the similarity of the photophysics between the aggregated domains in the PS blends and the 100% TTBP samples.

**Fig. 4:**
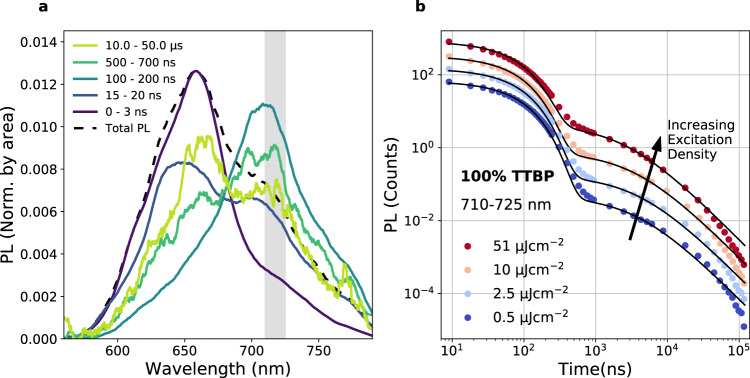
Time-resolved PL of TTBP. **a** Time-cuts of time-resolved photoluminescence for a 100% TTBP dropcast film at room temperature with an excitation fluence of 25 µJ cm^−2^, showing the early dominance of the 660 nm peak, followed by a red-shift to 720 nm over ~30 ns, followed by a shift back to 660 nm after ~500 ns. Grey bar indicates wavelength range in **b**. **b** Filled circles indicate PL signal integrated over the wavelength range indicated in grey in **a** with varied fluence. A relative increase in the delayed photoluminescence (after 500 ns) is seen as excitation density increases. Solid lines are fits consisting of the sum of a monomolecular decay (*τ* ~ 65 ns) and a bimolecular decay at later times (see Supplementary Note 2 for details).

For a singlet fission material, the photoluminescence yield of TTBP is unusually high. The PLQE is 20% in the solid state at room temperature, rising to 97% in increasingly dilute PS blends (Supplementary Fig. 5). In pure TTBP, there are two dominant spectral features, with distinct kinetics, shown in Fig. 4a. Immediately after photoexcitation, the emission is dominated by a feature which peaks at 660 nm with a shoulder at 600–620 nm. After delays of 100–500 ns, the 660 nm peak reduces in intensity to reveal a feature at 720 nm. When the time-cut spectra are normalised by area in Fig. 4a, there is an isoemissive point at 685 nm, indicating that these are two distinct species. The spectrum continues to shift until 1 µs after excitation, beyond which the spectrum is a constant mixture of the initial 660 nm peak and the 720 nm peak that dominates in the intermediate regime.

Although there is some early-time singlet–singlet annihilation (evident from the sub-linear increase in PL with excitation density and the difference in the initial PL decay kinetics with excitation density shown in Supplementary Fig. 6), by 10 ns this effect is negligible. Therefore, the PL emission rate at 10 ns can be used to benchmark the population of triplet pairs. From 10 ns to 500 ns the photoluminescence decay is monomolecular, with a lifetime of 65 ns and negligible excitation density dependence. Subsequently, as the pairs dissociate or decay radiatively, by 1 µs the photoluminescence is dominated by non-geminate TTA. The emission rate for non-geminate TTA is proportional to the square of the triplet population, and the decay is bimolecular. The delayed contribution is excitation density-dependent, with a stronger delayed component at higher excitation densities relative to the intensity of the early photoluminescence, characteristic of non-geminate, bimolecular TTA (Fig. 4b). Beyond ~50 µs, the PL response falls below this bimolecular fit, likely owing to time-dependent triplet mobility, with lower mobility triplets dominating at very long delays. The blue shift to the spectrum at late times can be understood as lower mobility triplets undergoing TTA with weak inter-triplet exchange coupling. We verify the linear and quadratic dependence of the integrated counts from each part of the fits with respect to the population density at 10 ns. Confirming the assignment, the delayed, bimolecular component scales with a power of 1.6 to the counts at 10 ns, whereas the monomolecular component scales with a power of 0.9 (Supplementary Fig. 7).

The photoluminescence yield increases as temperature decreases (Supplementary Fig. 10). We consider that this occurs owing to the suppression of pair separation as evidenced by the trESR, where fewer free triplets are observed at lower temperatures (Supplementary Fig. 22). The temperature dependence of photoluminescence (see above, Fig. 1c) in TTBP is strikingly similar to tetracene^14^, an indicator that the relative energy of singlet and triplet pair is similar in both materials: isoenergetic or narrowly exothermic to reach the intermediate pair-state, whereas separation of the pair state to free triplets is the endothermic step. At low temperatures, the photoluminescence yield increases and the spectrum blueshifts and narrows. Below 50 K, we see superradiance, as in tetracence^14^. At 11 K, the 720 nm peak is greatly reduced. This part of the spectrum has the strongest non-geminate TTA (as discussed in Supplementary Figs. 31–33). We, therefore, attribute the relatively low emission at 720 nm at 11 K to a lower population of free triplets available to annihilate, in combination with the superradiant enhancement of radiative rate from the singlet state. We note that it is also possible that at lower temperatures, the suppression of triplet hopping prevents the evolution of the pairs to geometries that emit at this low energy peak.

**Fig. 5:**
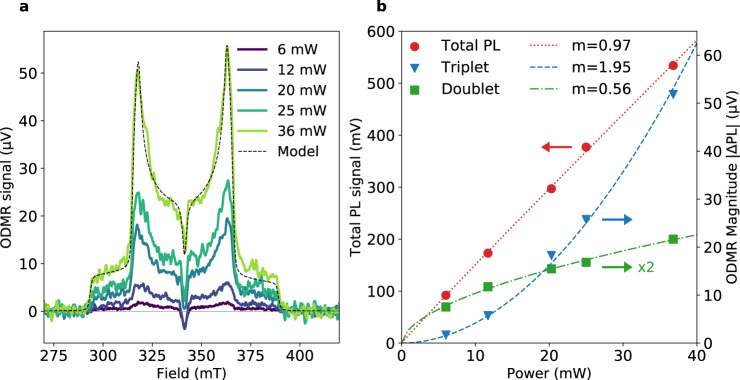
ODMR of TTBP. **a** ODMR of 100% TTBP film at 50 K using a 532 nm CW laser excitation at a range of excitation densities, labelled by the total laser power incident on the microwave cavity. The microwaves were modulated in a square wave at *f*_M_ = 315 Hz and data collected using an avalanche photodiode and lock-in amplifier. The in-phase signal is shown, and the out-of-phase signal was negligible in comparison. Black dotted line shows a simulation for a triplet signal performed with EasySpin^51^ + a Lorentzian radical signal, with the same zero-field splitting parameters for the triplet as used for the trESR simulation (*D* = 47.9 mT, *E* = 1.0 mT) and a linewidth of 1.3 mT. **b** Power dependence of total PL and ODMR (triplet and doublet features) for the same powers as used in **a**. Total PL is in red, left axis, circles; ODMR triplet peak ΔPL (366 mT) is in blue, right axis, triangles; doublet due to radicals (342 mT) is in green, right axis, squares. The lines are fits of PL or ΔPL ∝ (*incident power*)^*m*^ with *m* = 0.97 for the total PL (red dotted line), *m* = 1.95 for the triplet ODMR peak (blue dashed line), and *m* = 0.56 for the ODMR doublet signal (green dash-dot line). The fits demonstrate that they are in the linear, quadratic, and sub-linear regimes, respectively.

Figure 5 reports optically detected magnetic resonance (ODMR) with the effect of microwave absorption/emission detected via changes to the photoluminescence. This gives a very sensitive handle to explore the photoluminescence that results from paramagnetic species, especially triplets^43^. At 50 K, we observe a characteristic triplet spectrum in-phase with the microwaves, which matches the magnetic field positions of the trESR signal. The signal is positive, indicating an increase in photoluminescence with microwaves relative to microwaves off. This polarisation is consistent with thermalisation of the populations in the triplet sublevels, i.e., a Boltzmann distribution, as observed in ODMR of non-geminate TTA in TIPS-tetracene^44^.

The main peaks scale quadratically with excitation density, indicating that they arise from a bimolecular process—i.e., non-geminate TTA. This power dependence demonstrates that there is a radiative bimolecular decay pathway. We note that the central negative transition is consistent with a sub-population of radical doublets undergoing triplet-radical annihilation (TRA), scaling sub-linearly with excitation density, indicating that TRA is not the dominant loss process. The observed doublet signal is consistent with a small degree of charge formation and accumulation (signal magnitude varied slowly with light exposure over time—see Supplementary Fig. 34). The doublet peak has a small integrated area in comparison with the main peaks—indicating only a minor contribution from TRA relative to TTA.

By varying the microwave modulation frequency *f*_*M*_, we can learn about the characteristic timescale of TTA (Supplementary Fig. 30). Forty percent of the non-geminate TTA occurs on a timescale of ~100 µs, whereas the remaining 60% occurs on a timescale faster than 3 µs, which we could not resolve owing to the frequency limitations of the lock-in amplifier. The 100 µs component occurs on a timescale that we assume, from the trESR (Supplementary Fig. 27), is longer than the spin-lattice relaxation time *T*_*1*_, meaning the sublevels will be occupied in a Boltzmann distribution, giving a positive ODMR signal from non-geminate TTA. However, the contribution from a timescale faster than 3 µs, which is faster than the estimated *T*_*1*_ (on the order of 10 µs—Supplementary Table 3), should arise from a non-Boltzmann population of the triplet sublevels as in the trESR (Supplementary Fig. 27) we observe a spin polarised triplet up to 15 µs in time.

Long-pass and short pass filters allowed coarse spectral resolution, and we confirmed that the lowest energy peak has a stronger contribution from TTA than the higher-energy peak (See Supplementary Figs. 31–33). This is in agreement with the time-resolved photoluminescence, where the delayed spectrum is more red-shifted than the prompt photoluminescence. We consider that the distinct spectral feature at 720 nm could be due to a specific geometric orientation of a pair of TTBP molecules. It has been shown in TIPS-tetracene that emission from triplet pairs with strong exchange coupling is red-shifted relative to that of weak coupling^45^, so this feature could be attributed to very strongly coupled pairs. This would likely originate in pairs of molecules that are slip-stacked face-to-face at only 0.35 nm, consistent with the crystal structures reported by Müller et al.^35^, notably closer than any pair in the TIPS-tetracene crystal structure^45^.

Through a combined analysis of the TA, time-resolved photoluminescence, trESR and ODMR, we have built a detailed picture of the photophysics, where triplet pairs are formed rapidly in quantitative yield and later separate by a temperature-activated process. We have shown efficient radiative decay of triplet pairs as singlet excitons through geminate TTA of bound pairs, or through non-geminate TTA of free triplets.

The unique photophysics of TTBP are particularly well-suited for the thermodynamic requirements of enhancing photovoltaics by singlet exciton fission. The general scheme for such an enhancement is to arrange that triplet exciton pairs photogenerated in a wider gap organic semiconductor can be transferred to a lower gap inorganic semiconductor solar cell, where they each generate electron-hole pairs. Schemes developed in the literature include triplet transfer to luminescent PbS quantum dots that generate photons that can be absorbed by a silicon cell^3^ or by the direct energy transfer of the triplet exciton through an interface with silicon^7^. Endothermic fission maximises the energy of the triplets produced, and also ensures that interconversion of singlet excitons and triplet exciton pairs is reversible. In a single film, as studied in this work, where triplets are not extracted to a lower gap solar cell (equivalent to “open circuit” conditions), the only available radiative decay channel will be through TTA (noting that phosphorescence from triplets in hydrocarbon semiconductors is strongly forbidden). We think of the singlet fission system as a black box: photons enter the system, and in a single film (“open circuit” operation) where no triplet excitons are extracted, 100% efficiency of photons being re-emitted would indicate the highest possible operating efficiency with zero non-radiative losses^46^. In the analogous situation for charges in silicon or GaAs, non-radiative voltage loss is described by Ross as $- \frac{{kT}}{q}ln(\varphi )$, where *φ* is the electro- or photoluminescence quantum yield^47–49^. *φ* must be maximised to eliminate non-radiative charge recombination in a single-junction solar cell. For singlet fission, *φ* must be maximised to eliminate non-radiative triplet decay pathways. We propose that the high PLQE of TTBP provides an important direction for the future development of singlet fission solar cells. A further virtue of the TTBP system is a remarkable improvement in stability we find under illumination in ambient conditions compared with standard linear acenes (Supplementary Fig. 2 shows the degradation relative to that of a representative solution-processable acene derivative, TIPS-tetracene). In combination with the high luminescence yield, this provides a very attractive route to enhance the efficiency of solar cells.

## Methods

### Sample preparation

TTBP was synthesised as described by Müller et al.^35^. Films were deposited on untreated glass by dropcasting from 50 mg mL^−1^ solutions in 1,2-dichlorobenzene. Blends with polystyrene were also dropcast from solutions of mixed 190 kDa polystyrene and TTBP in a mix of 50:50 toluene and 1,2-dichlorobenzene. All solvents were used as received from Sigma-Aldrich. Samples were encapsulated in a nitrogen atmosphere using polyisobutene and a glass slide before exposing to air.

### Steady-state optical measurements

Absorption spectra were taken with an Agilent 8453 UV-visible spectrometer. Steady-state photoluminescence measurements were recorded on an Edinburgh Instruments FLS980 fluorimeter. PLQE was measured using a 520 nm CW laser and the sample inside an integrating sphere, with detection via a fibre optic cable to an Andor iDus DU420A BVF Si detector. PLQE was calculated by the de Mello method to account for secondary absorption of scattered light^50^. For photoluminescence measurements in a magnetic field, we used the CW 520 nm source and a pair of lenses to project the photoluminescence emitted to a solid angle of 0.1*π* onto an InGaAs detector, with the sample positioned in an electromagnet (GMW—Model 3470) with 3 cm distance between cylindrical poles. The magnetic field for a given voltage supplied to the electromagnet was measured with a gauss metre. Photostability measurements were performed using the same detector and excitation, with the transmitted 520 nm laser signal being measured over time, and converted to absorption using *Absorbed photons* = *Incident photons*−*Transmitted photons*.

### Time-resolved photoluminescence

Time-resolved and temperature-dependent photoluminescence were recorded using an electrically gated intensified charge-coupled device (ICCD) camera (Andor iStar DH740 CCI-010) connected to a calibrated grating spectrometer (Andor SR303i). Pulsed 500 nm photoexcitation was provided at a repetition rate of 1 kHz from a home-built non-collinear optical parametric amplifier (NOPA) powered by a Ti:sapphire amplifier (Spectra Physics Solstice Ace) that generated 100 fs duration pulses centred at 800 nm. A 550 nm long-pass filter (Thorlabs) was used to prevent scattered laser signal from entering the camera. Temporal evolution of the PL emission was obtained by stepping the ICCD gate delay with respect to the excitation pulse. The minimum gate width of the ICCD was 3 ns. For temperature-dependent measurements, the sample was mounted in a helium cryostat and kept under high vacuum (10^−5^ mbar).

### Transient absorption

TA formed on two home-built TA setups, one for fs–ps timescales and one for ns–µs timescales^5^. In both systems, the sample is photoexcited by a narrow pump pulse. The pump is followed by a broadband probe pulse, at a controlled delay, using a mechanical delay stage (Newport) for fs–ps timescales or an electronic delay generator (Stanford Research Systems DG645) for ns–µs timescales.

For fs–ps TA, a light conversion PHAROS laser system with 400 μJ per pulse at 1030 nm with a repetition rate of 38 kHz was used. The output is divided; one part was focused on a 4 mm YAG substrate to produce the continuum probe beam from 520 to 950 nm. The second part of the PHAROS output was fed into a narrow band optical parametric oscillator system (ORPHEUS-LYRA, light conversion) outputting the pump beam at 550 nm. The on–off pump pulses were generated by means of a mechanical chopper (Thorlabs) before hitting the sample. The pump and probe beams were focused with sizes 300 × 300 µm and 130 × 130 µm, respectively, at the sample position. The probe pulse transmitted through the sample was collected using a silicon line scan camera (AViiVA EM2/EM4) with a visible monochromator 550 nm blazed grating.

For ns–µs TA, two configurations were used. In the first, used for the solid-state samples, the pump was generated by the second harmonic (532 nm) of a Q-switched Nd:YVO4 (1 ns pump length, Advanced Optical Technologies Ltd AOT-YVO-25QSPX). The probe was generated by home-built broadband visible (500–770 nm) NOPA, pumped using the 400 nm second harmonic of the Ti:sapphire amplifier (Spectra Physics Solstice Ace). In the second configuration, used for the solutions and sensitisation, the pump was the 400 nm second harmonic of the Ti:sapphire amplifier, whereas the probe was generated by a LEUKOS Disco 1 UV low timing jitter supercontinuum laser (STM-1-UV). This laser produces pulses with a temporal breadth below 1 ns from 200 to 2400 nm. In both configurations, the probe is split by a 50% reflectance beam splitter to create a reference. The pump and probe beams are overlapped on the sample adjacent to the reference beam. This reference is used to account for any shot-to-shot variation in transmission. The transmitted probe pulses were collected with a silicon dual-line array detector (Hamamatsu S8381-1024Q), which was driven and read out by a custom-built board from Stresing Entwicklungsbüro.

### Numerical methods

We use a genetic algorithm to deconvolute the overlapping TA spectral signatures of individual excited states and obtain their kinetics.

### Time-resolved ESR

Transient ESR was performed using a Bruker MD5 dielectric ring resonator and an X-band ESR spectrometer. Pulsed 532 nm excitation was provided by a Nd:YAG laser (Atum Laser Titan AC compact 15 MM) with a 5 ns pulse length (the laser beam was depolarised before excitation of the sample). Continuous-wave microwave irradiation was applied as the static magnetic field was swept.

### Optically detected magnetic resonance

Optically detected magnetic resonance was performed using a home-built optical resonator (based on a dielectric ring resonator), which allows for excitation of the sample and collection of photoluminescence in transmission mode). A 532 nm diode-pumped solid-state laser (DJ532-40 Thorlabs) was used for continuous excitation of the sample (the laser beam was depolarised before excitation of the sample). The integrated photoluminescence was collected by a silicon avalanche photodetector (APD120A Thorlabs) while the laser line was removed via a 532 nm notch filter (Semrock). The ODMR was carried out at X-band and the microwaves were square-wave modulated at frequencies between 17 Hz and 100 kHz. The change in photoluminescence owing to microwave absorption was monitored at the microwave modulation frequency using lock-in detection (Stanford Research Systems SR830) as the static magnetic field was swept through resonance.

## Supplementary information

Supplementary Information

## Data Availability

The data sets generated during and/or analysed during the current study are available in the University of Cambridge data repository at 10.17863/CAM.63436
