# Supplementary material for: Singlet exciton fission in a modified acene with improved stability and high photoluminescence yield
Source: Nat Commun. 2021 Mar 9;12:1527. doi: 10.1038/s41467-021-21719-x (PMC7943798; doi:10.1038/s41467-021-21719-x)
Supplement: Supplementary file 1 — Supplementary Information [file 41467_2021_21719_MOESM1_ESM.pdf]

Singlet Exciton Fission in a Modified Acene with Improved Stability and High Photoluminescence Yield

Peter J. Budden, Leah R. Weiss, Matthias Müller, Naitik A. Panjwani, Simon Dowland, Jesse R. Allardice, Michael Ganschow, Jan Freudenberg, Jan Behrends, Uwe H. F. Bunz, Richard H. Friend

Supplementary Information

Table of Contents

1) Comparison to TIPS-tetracene 2
2) Photoluminescence 3
3) Transient Absorption 9
4) Triplet Sensitisation and Yield 10
5) Magnetic Field Effect on Photoluminescence 14
6) trESR 17
7) ODMR 24

Supplementary Note 1: Comparison to TIPS-tetracene

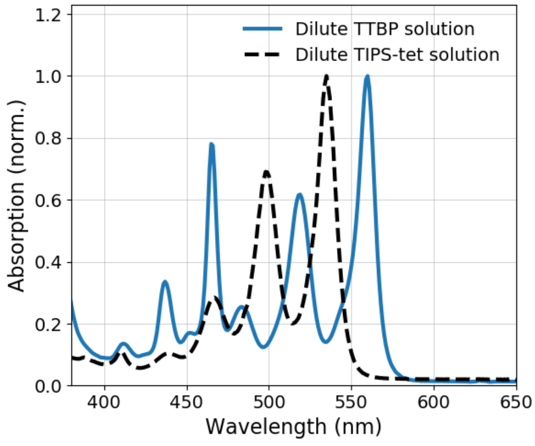

Supplementary Figure 1: UV-vis absorption spectra for TTBP (blue solid line) and TIPS-tetracene (black dashed line), showing their similar band-gap/absorption onset.

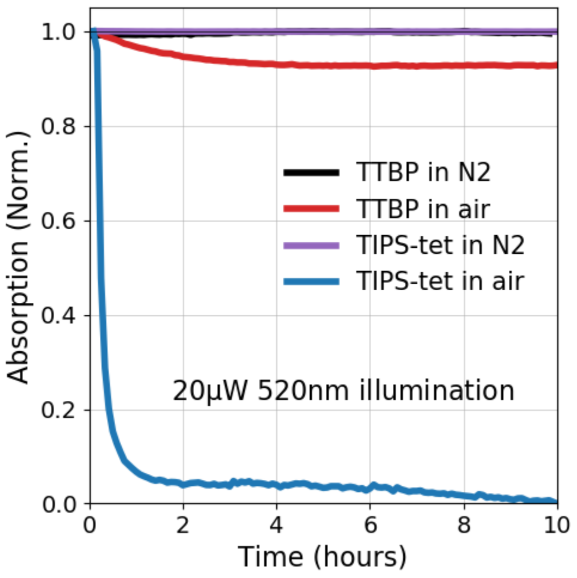

Supplementary Figure 2: Stability of TTBP compared to TIPS-tetracene. Dropcast samples prepared from 50 mg mL^{-1} solutions in DCB were illuminated by 20 µW CW 520 nm, with the absorption monitored over time. Black and purple lines show samples encapsulated in an O_2 and H_2O free environment, while red and purple lines show unencapsulated films in ambient conditions.

The stability of TTBP in air is shown to be superior to TIPS-tetracene in Supplementary Figure 2. Under CW illumination, there is a small initial reduction of absorption, likely due to surface damage, after which there is no further degradation. In contrast, TIPS-tetracene absorption falls by 90% within the first hour of illumination and goes on to decay to 0 over 10 hours. As well as dropping by only 6% compared to 90%, the initial decay in TTBP is much slower, taking 4 hours compared to 1 hour.

Both light and the presence of O_2 and/or H_2O are required for the samples to degrade, as we observed little to no degradation of well-encapsulated samples in an inert N_2 atmosphere. For the stability test, the spot size was ~0.8 mm in diameter, giving an irradiance of ~1 $mWcm^{-2}$

Supplementary Note 2: Photoluminescence

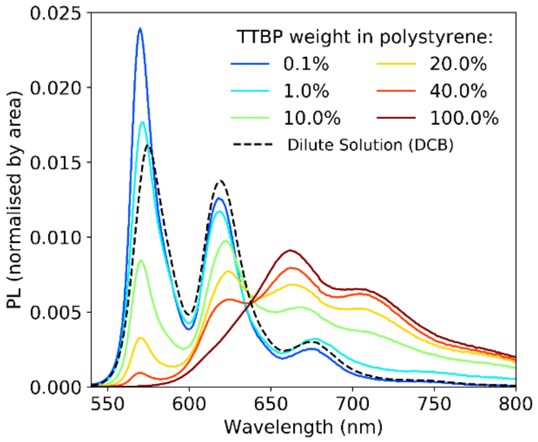

Supplementary Figure 3: Steady state photoluminescence of TTBP in PS at varying concentrations from 100% TTBP (dark red) to 0.1% (blue), with excitation from a Xenon lamp with a monochromator set to 530 nm. Dilute solution photoluminescence spectrum, with dichlorobenzene (DCB) as solvent, is shown in dashed black.

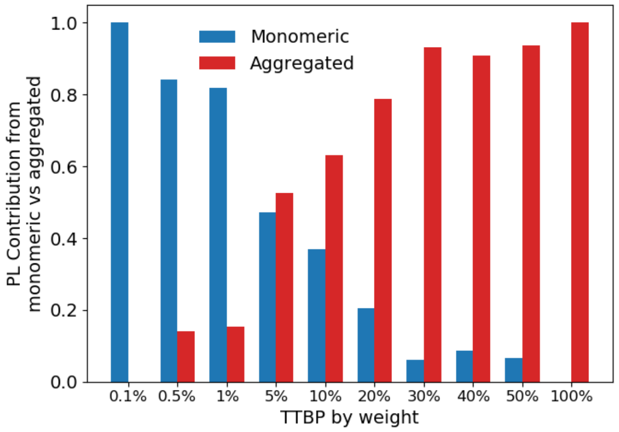

Supplementary Figure 4: Contribution to the steady-state PL spectrum from the monomeric spectrum (=0.1% TTBP in PS) vs aggregated spectrum (=100% TTBP).

The photoluminescence spectra of the different concentration TTBP/PS blends can be used to quantify the degree of aggregation within the films. In Supplementary Figure 3, it is clear that there are contributions from two distinct spectra – the aggregated TTBP with broad peaks at ~660 nm and ~710 nm, and the monomer TTBP spectra which closely matches that of the dilute solution, characterised by narrower peaks at 570 nm, 620 nm, and 675 nm. Apart from some modulation of the relative intensities of the vibronic peaks in the monomer spectrum, the spectra of the PS blends can be recovered from a linear combination of the 0.1% and 100% spectra. The relative contribution from these two spectra at each concentration, using a simple linear regression fit, is shown in Supplementary Figure 4. Importantly, as shown in Supplementary Figure 5, the PLQE increases by a factor of 5 between the aggregate and the dispersed TTBP, implying that even in the 5% TTBP film where there is roughly even contributions to the PL, ~5 times as much of the excitation photons are absorbed by the aggregated TTBP than by the dispersed.

This analysis of the photoluminescence reveals a small fraction of monomeric molecules in the intermediate concentration blends that are not aggregated, but the majority have identical luminescence to the 100% film. This is as we expect due to the low affinity between

PS and the strongly crystallising molecular material. At intermediate concentrations of TTBP, there will only be limited solubility for isolated TTBPs, beyond which we see phase separation and TTBP domains forming with the same optical properties as the 100% TTBP film.

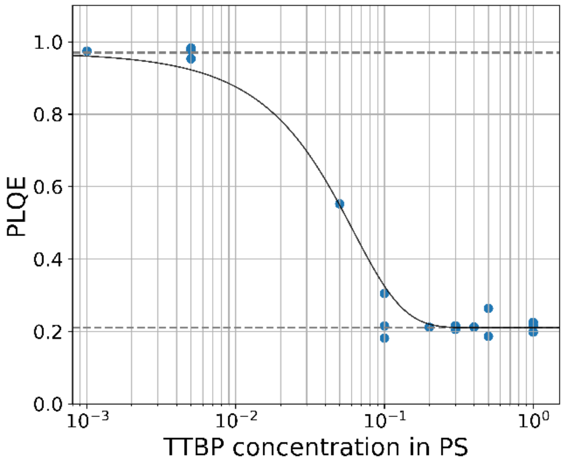

Supplementary Figure 5: PLQE for a range of concentrations in PS, showing that as TTBP aggregates, PLQE plateaus at 20%.

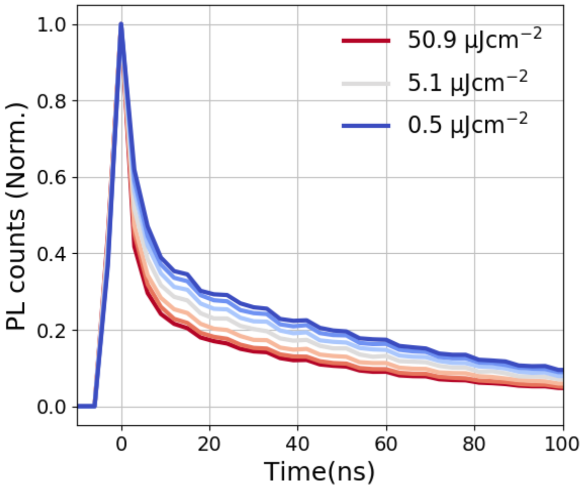

Supplementary Figure 6: Early time kinetics of the photoluminescence of 100% TTBP at room temperature at 710-725 nm. Excitation is by a 200 fs, 500 nm pulse at 1 kHz, while the smallest time step and instrument response function is ~3 ns.

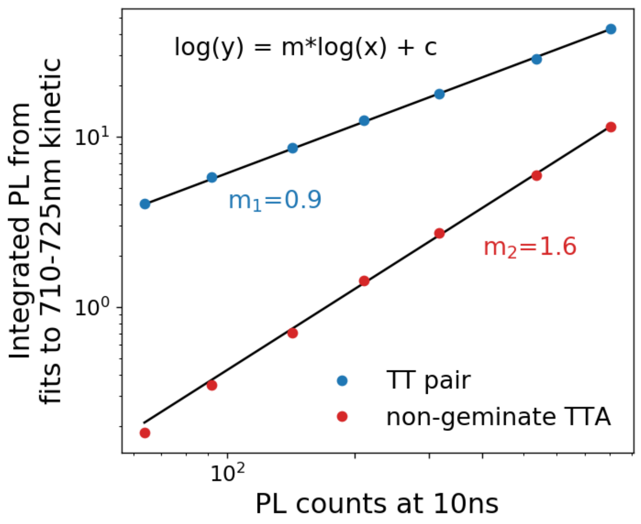

Supplementary Figure 7: Dependence of the integrated intensity of the two temporal components of the 710-725 nm PL kinetic on excitation density. Excitation density of triplet pairs at 10 ns is proportional to the PL signal at 10 ns. PL from triplet pairs, the monomolecular part of the decay, increases approximately linearly with density of triplet pairs at 10 ns ($m = 0.9$). The bimolecular part of the decay, from non-geminate TTA, increases super linearly ($m = 1.6$).

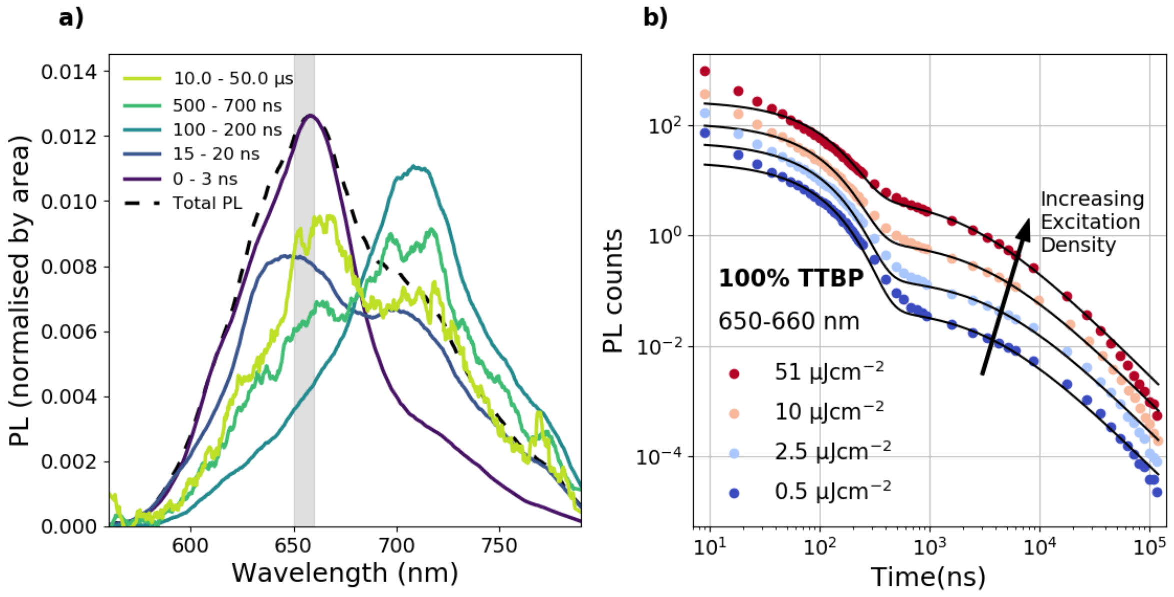

Supplementary Figure 8: PL kinetics at 650-660 nm, presented as in Figure 4 of the main text where 710-725 nm is highlighted. Before ~20 ns, there is some prompt singlet PL which causes the fit to the geminate/non-geminate TTA model to under-predict the actual signal.

In the models for the PL kinetics shown above in Supplementary Figure 8, and in Main Text Figure 4b, the initial decay is attributed to geminate triplet pairs, and is fitted with a single exponential with a lifetime of ~65 ns. The delayed components of the kinetics are fitted using a non-geminate TTA model. We begin with the model for the time evolution of triplet population, $[T]$, from Poletayev et al.[1], Supplementary Equation 2.1, where $G(t)$ is the generation of triplets over time, τ is the monomolecular decay lifetime and γ is the bimolecular decay constant.

$$\frac{d[T]}{dt} = G(t) - \frac{[T]}{\tau} - \gamma[T]^2 \qquad 2.1$$

At delayed times, we assume the generation of triplets is zero. Then, solving the differential equation for $[T]$ gives the solution in Supplementary Equation 2.2

$$[T] = \frac{\left(\frac{1}{\tau}\right) e^{\frac{c_1}{\tau}}}{e^{\frac{t}{\tau}} - \gamma e^{\frac{c_1}{\tau}}} \qquad 2.2$$

Setting $PL \propto [T]^2$, and allowing both τ and γ to vary in this model, we found that τ was always very large, and hence the contribution from monomolecular decay in our fit was negligible in comparison to bimolecular decay. This further simplifies the model to Supplementary Equation 2.3, for which the solution is given in Supplementary Equation 2.4.

$$\frac{d[T]}{dt} = -\gamma[T]^2 \qquad 2.3$$

$$[T] = \frac{1}{\gamma t + c} \qquad 2.4$$

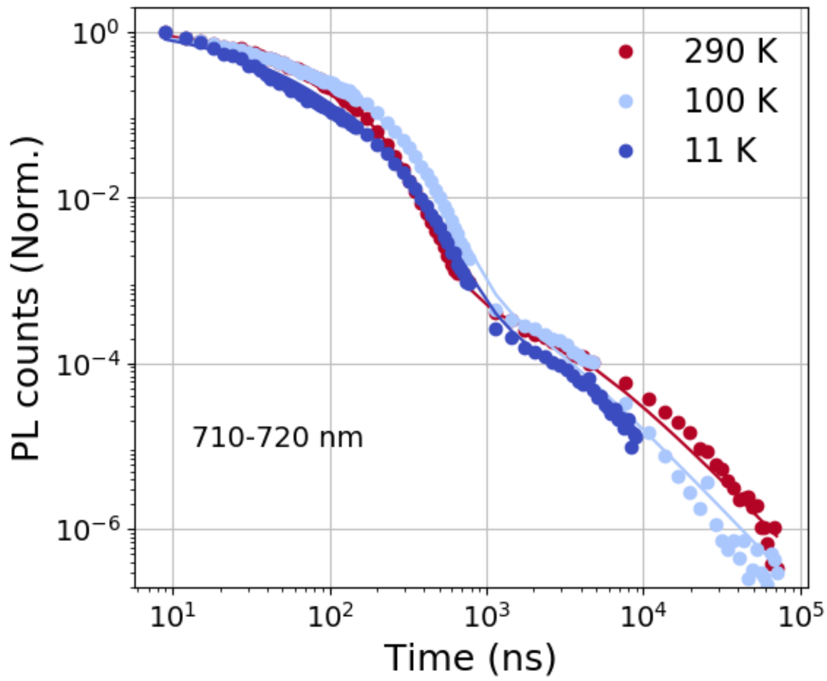

Supplementary Figure 9: PL kinetics for 100% TTBP, excited at 500 nm with 200 fs pulses, at 710-725 nm, for a range of temperatures. Kinetics are normalised to counts at 3 ns. Solid lines are fits to a stretch exponential plus bimolecular decay model, detailed below. 11 K measurement was not extended beyond 10 μs due to low signal/noise.

Supplementary Figure 9 shows PL kinetics at different temperatures at 710-725 nm. At 100 K, the geminate triplet pair PL, which dominates from 10 – 500 ns, decays slower than at room temperature ($\langle\tau\rangle$ = 73 vs 58 ns). The triplet pairs dissociate more slowly at lower temperatures. At 11 K, the superradiance effect accelerates the radiative rate of the singlet

and/or geminate pair, leading to a faster decay on this timescale. Beyond 1 μs, at lower temperatures there are fewer free triplets and consequently less non-geminate TTA.

Similarly as for the room-temperature fluence-dependent fits in Main Text Figure 4b and Supplementary Figure 8, the fits in Supplementary Figure 9 comprise two components, one for the geminate triplet pair emission and a second for delayed, non-geminate TTA. While at room temperature, a singlet exponential adequately fits the triplet pair emission kinetic, with a lifetime of 65 ns, this does not accurately describe the behaviour at low temperatures. Instead, we use a stretch exponential, Supplementary Equation 2.5, which approximates a distribution of decay timescales across the population.

$$PL(t) = PL(0)e^{-\left(\frac{t}{\tau_K}\right)^{\beta}} \qquad 2.5$$

The area under a stretched exponential can be interpreted as the mean relaxation time, $\langle\tau\rangle$, expressed in Supplementary Equation 2.6 in terms of the Gamma function, Supplementary Equation 2.7.

$$\langle\tau\rangle = \int_0^\infty dt\, e^{\left(-\frac{t}{\tau_K}\right)^{\beta}} = \frac{\tau_K}{\beta}\Gamma\left(\frac{1}{\beta}\right) \qquad 2.6$$

$$\Gamma(z) = \int_0^\infty t^{z-1}e^{-t}dt \qquad 2.7$$

The mean relaxation times for the different temperatures are shown in Supplementary Table 1. The delayed, non-geminate TTA is fitted to the same model as the room-temperature kinetics in Main Text Figure 4b and Supplementary Figure 8, using Supplementary Equation 2.4. This model of a single bimolecular decay constant is not as good a fit at lower temperatures, likely due to greater trapping of triplets and therefore greater variability of triplet mobility over time.

Temperature (K)	$\langle\tau\rangle$ (ns)
290	58
100	73
11	34

Supplementary Table 1: Mean relaxation times for a stretch exponential fit to the geminate triplet pair emission decay at different temperatures.

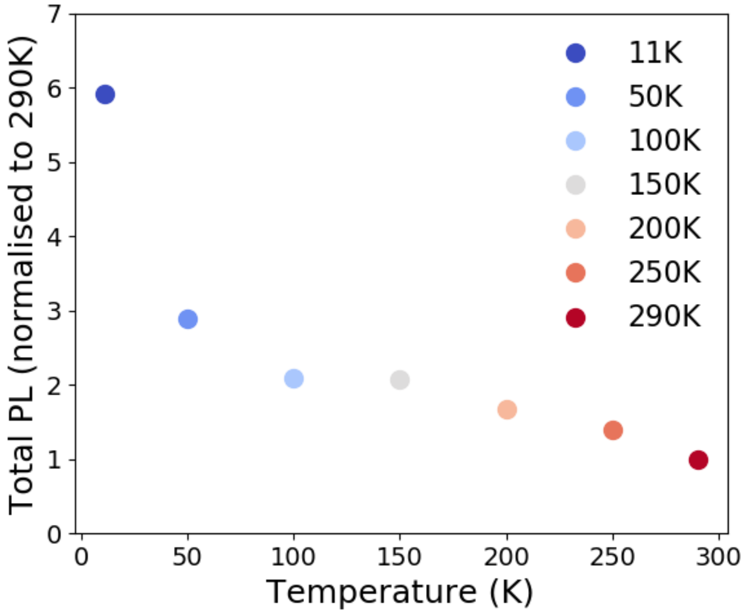

Supplementary Figure 10: Temperature dependence of the time-integrated and wavelength-integrated photoluminescence of 100% TTBP, excited at 500 nm with 200 fs pulses at 1 kHz, normalised to the value for 290 K. The sample spot may change slightly between different temperatures.

Supplementary Note 3: Transient Absorption

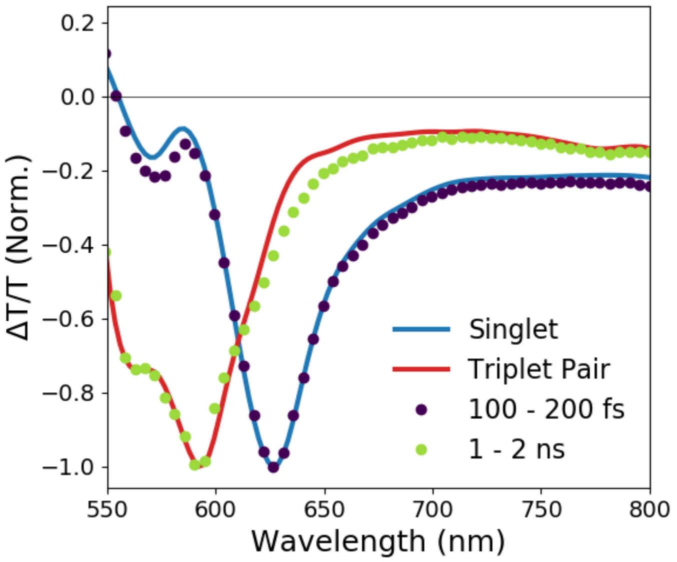

Supplementary Figure 11: Genetic Algorithm analysis. Solid lines: Species associated spectra from the Genetic Algorithm analysis of ps-resolved transient absorption of a 7% TTBP in PS film, for which kinetics are shown in Main Text Figure 2c. Dots: transient absorption data at very early time, 100-200 fs (dark blue dots) and at 1-2 ns (green dots).

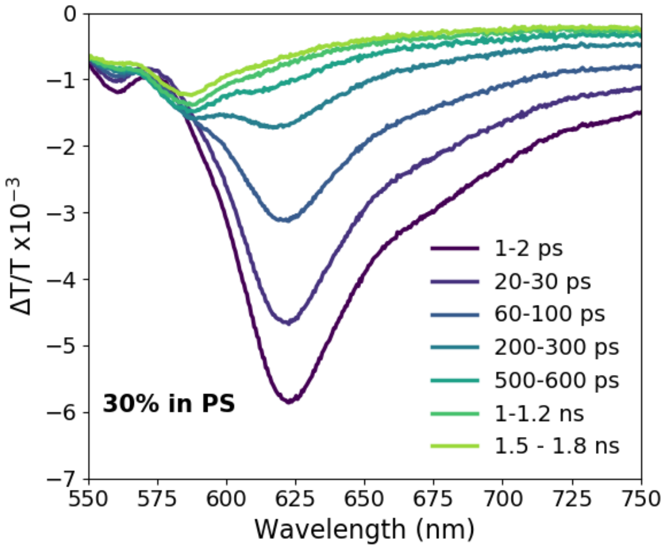

Supplementary Figure 12 ps-resolution TA of 30% TTBP in PS film, showing broadly similar behaviour to 7% in PS.

Supplementary Note 4: Triplet Sensitisation and Yield

To reveal the absorption spectrum of the TTBP triplet exciton, a triplet sensitizer, *N-methylfulleropyrrolidine* (NMFP), was used. NMFP undergoes rapid intersystem crossing (ISC) from S_1 to T_1 and has a triplet energy higher than that of TTBP, ensuring that triplet energy transfer to TTBP is energetically favourable. A mixed solution of NMFP and TTBP was prepared. From comparison of the UV-vis absorption of the sensitization solution to the respective stock solutions at 1 mg mL^{-1} we confirmed the relative concentrations of NMFP and TTBP to be 1.1 mg mL^{-1} and 0.52 mg mL^{-1} respectively, see Supplementary Figure 13.

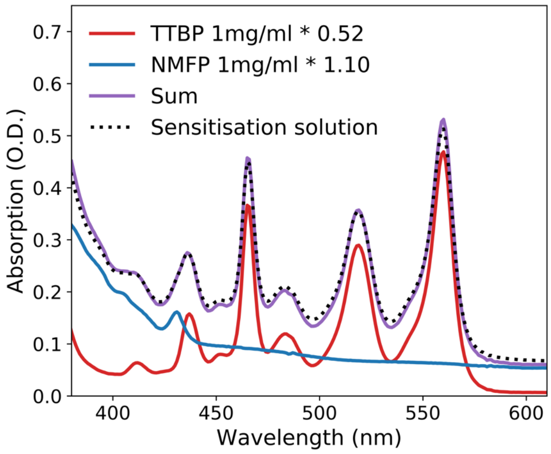

Supplementary Figure 13: UV-vis absorption for TTBP (red) and NMFP (blue) 1 mg mL^{-1} solutions, and a mixed solution of both TTBP and NMFP (dotted black), all in THF. The TTBP and NMFP absorption spectra are scaled by factors of 0.52 and 1.10 respectively, such that their sum (purple) matches the absorption spectrum of the mixed solution, with the scaling factors optimised using a least-squares regression method.

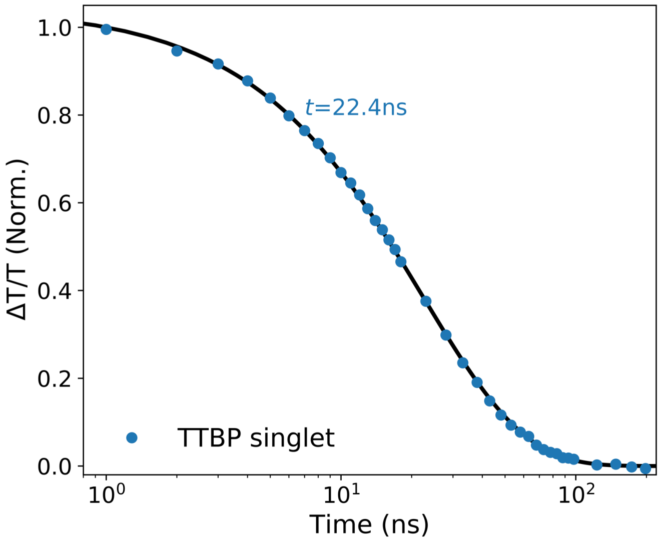

Supplementary Figure 14: Transient absorption kinetic at 630 nm for 1 mg mL^{-1} solution of TTBP in THF, with a mono-exponential fit. Excitation was performed with a 500 Hz 200 fs 400 nm pulse with a fluence of 500 µJcm^{-2}. The probe was a ~1 ns 1 kHz broadband probe from a supercontinuum laser, with the delay relative to the excitation pulse controlled electronically.

The mixed solution was measured with ns-resolution transient absorption, and the results compared to the pure solutions of dilute TTBP or NMFP. The singlet exciton of TTBP has a strong, sharp photoinduced absorption (PIA) at 630 nm. In a dilute solution, this decays with a lifetime of 22 ns

(Supplementary Figure 14) in agreement with the fluorescence lifetime in dilute solutions/films. NMFP, when excited at 400 nm, quickly undergoes intersystem crossing from the singlet, which has a broader PIA extending out into the near IR, to the triplet exciton, which has a PIA peaked at 700 nm and negligible absorption beyond 850 nm. In a pure solution of NMFP in THF, the lifetime of this triplet state was found to be 11.1 µs, implying a decay rate $k_{NMFP} = 9 \times 10^4$ s^{-1}. When mixed together and excited at 400 nm, TTBP singlets decay whilst the NMFP triplets transfer to the TTBP molecules when they collide, as the TTBP triplet is at lower energy than the NMFP triplet. This results in a quenching of the NMFP triplet signal from a lifetime of 11.1 µs to 1.3 µs. The triplet energy transfer rate, k_{TET} can then be calculated as 6.8 x 10^5 s^{-1}. Additionally, the lifetime of the triplets formed on TTBP is extracted as 9.4 µs, implying $k_{TTBP} = 1.2 \times 10^5$ s^{-1}.

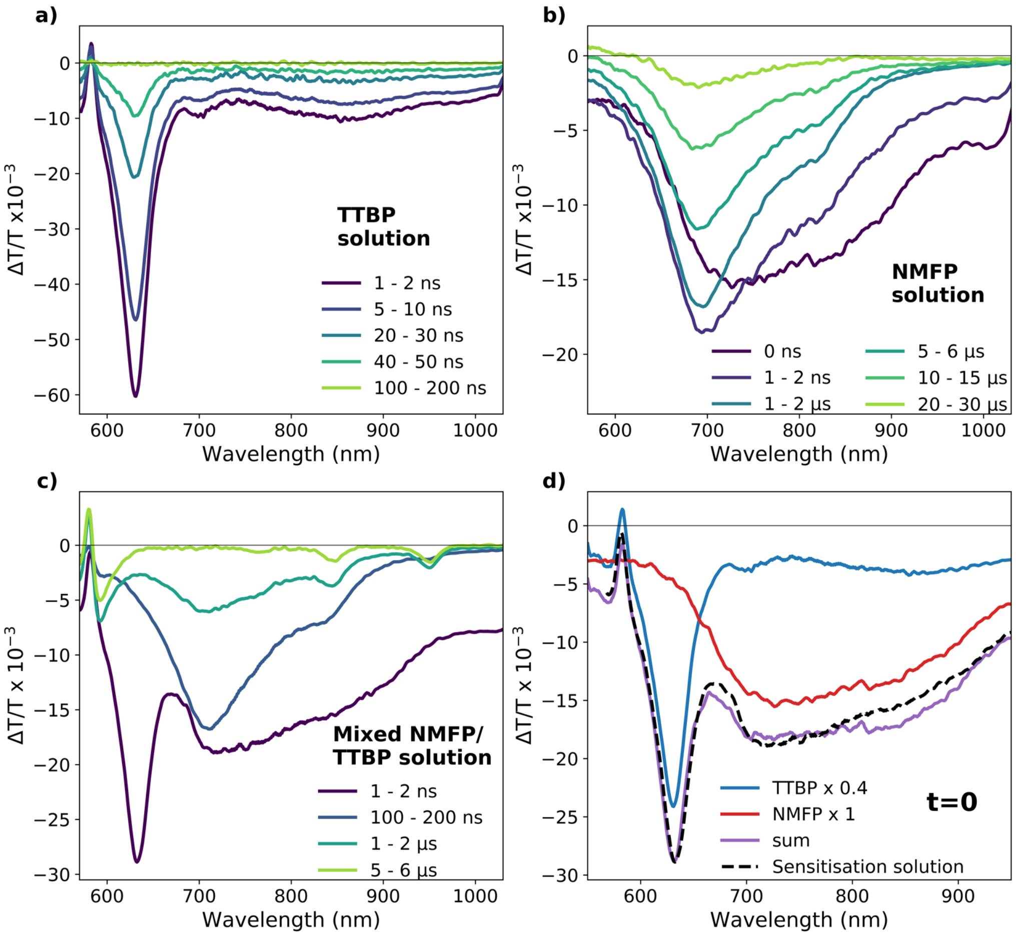

Supplementary Figure 15: Transient absorption of a) TTBP solution, b) NMFP solution, and c) mixed TTBP and NMFP solutions. d) Comparison of the spectra at t=0 showing the relative contributions from TTBP and NMFP to the mixed solution spectrum. Excitation was performed with a 500 Hz 200 fs 400 nm pulse with a fluence of 500 µJcm^{-2}. The probe was a ~1 ns 1 kHz broadband probe from a supercontinuum laser, with the delay relative to the excitation pulse controlled electronically.

In order to quantify the relative magnitudes of the singlet and triplet PIAs in TTBP, we need to understand what the relative population of TTBP triplets is at the peak of the triplet signal (~3 µs). Using the following scheme, we recreated the population dynamics based on the decay rates extracted from the TA data.

$$\begin{array}{ccc} & k_{\mathrm{TET}} & \\ \text{NMFP triplet} & \rightarrow & \text{TTBP triplet} \\ \downarrow k_{\mathrm{NMFP}} & & \downarrow k_{\mathrm{TTBP}} \end{array} \qquad (4.1)$$

Solving the coupled differential equations for the population of NMFP triplets (*N*) and TTBP triplets (*T*), gives the following solutions:

$$N = A_0 e^{-(k_{TET}+k_{NMFP})t} \quad (4.2)$$

$$T = A_0 \frac{k_{TET}}{k_{TET}+k_{NMFP}}\left(-e^{-(k_{TET}+k_{NMFP})t} + e^{-k_{TTBP}t}\right) \quad (4.3)$$

Inserting the rates from fitting the transient absorption kinetics in Supplementary Figure 16a, shows that the peak population of TTBP triplets is 53% of the initial population of NMFP triplets (Supplementary Figure 16b). We must also take in to account the ISC efficiency of NMFP, which is reported elsewhere to be 90-100%[2]. In this case we will use 90% as a lower bound.

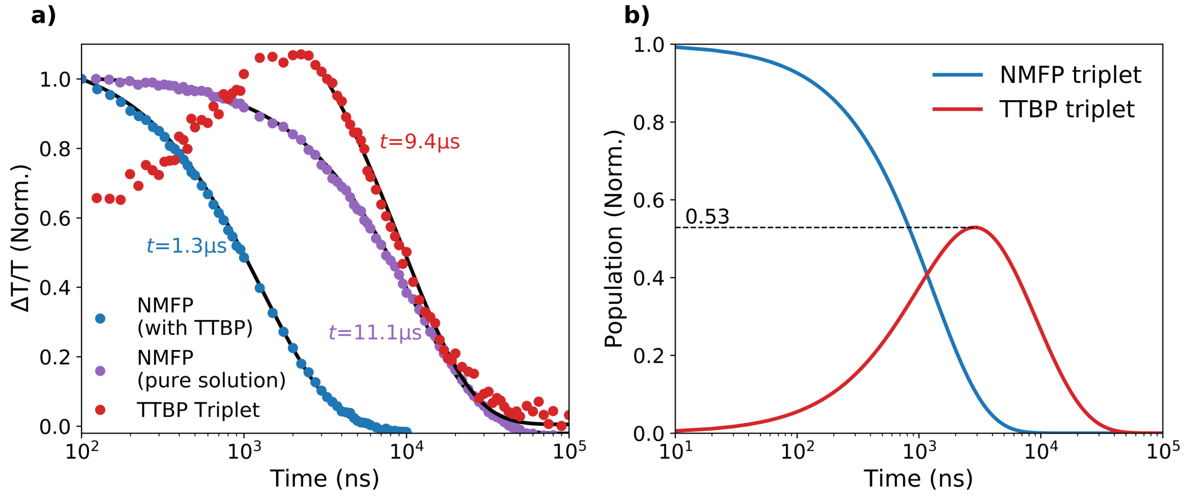

Supplementary Figure 16: a) Kinetics taken from the transient absorption spectra shown in Supplementary Figure 15: Transient absorption of a) TTBP solution, b) NMFP solution, and c) mixed TTBP and NMFP solutions. d) Comparison of the spectra at t=0 showing the relative contributions from TTBP and NMFP to the mixed solution spectrum. Excitation was performed with a 500 Hz 200 fs 400 nm pulse with a fluence of 500 μJcm-2. The probe was a ~1 ns 1 kHz broadband probe from a supercontinuum laser, with the delay relative to the excitation pulse controlled electronically.Supplementary Figure 15. Blue circles are NMFP with TTBP kinetic taken at 700 nm in the mixed solution. Purple circles are NMFP pure solution kinetic taken at 700 nm. Red circles are TTBP triplet kinetic taken at 930 nm in the mixed solution. Mono-exponential decay fits are made to each kinetic, plotted in black. b) Population kinetics in the mixed solution recreated utilising the lifetimes and triplet transfer rate extracted from a).

When excited at 400 nm, based on the summing of the absorption spectra in Supplementary Figure 13, we create singlets in TTBP and NMFP in a ratio of 0.04/0.21 = 0.21. Then we must include a factor of 0.9 for the NMFP ISC efficiency, and 0.53 for the population dynamics, to get a ratio between initial TTBP singlet population and peak TTBP triplet population of 0.43. At $t = 0$ in the sensitization solution, there is a small contribution at 630 nm from the NMFP (Supplementary Figure 15), which we subtract to leave a PIA of magnitude 2.4×10^{-3} from TTBP singlets. By comparison, the peak TTBP triplet PIA at 590 nm is 7.1×10^{-4}. Therefore, the relative magnitudes of the absorption cross-sections of the singlet 630 nm PIA to triplet 590 nm PIA is 7.8. It is not unusual to report much larger cross-sections for singlet PIAs than triplets, for example in TIPS-tetracene the ratio has been reported to be 26[3].

Although the sensitization measurement is performed in solution, we use the relative absorption cross-sections of the singlet and triplet to estimate triplet yield in solid films. The method of comparing the singlet and triplet absorptions from a singlet measurement offers a more reliable and self-consistent estimate of yield than using excitation density to benchmark the initial population of singlets, which would have to be done twice with completely different excitation sources for the film

measurement (fs OPA excitation) and sensitization (1 ns 532 nm Nd:YAG excitation). Importantly, it also cannot account for heterogeneity in optical density in the films, whereas the method used here avoids this issue. Inherent in any solution sensitization method is the difference between solution and solid state, which likely introduces a systematic error.

Fluence (μJcm^{-2})	Singlet PIA (100 fs)	Triplet PIA (1 ns)	Triplet yield
25	-9.70E-03	-2.49E-03	201%
8	-3.09E-03	-1.04E-03	265%
4	-6.68E-04	-2.86E-04	335%

Supplementary Table 2: Triplet yield calculated for the 3 fluences shown in Supplementary Figure 17, using the relative absorption cross sections for singlet and triplet calculated from solution sensitisation.

Supplementary Table 2 shows the magnitudes of the singlet and triplet PIAs and the resulting calculated triplet yield for a range of fluences in a 7% TTBP in PS film. We see that across a range of excitation densities the calculated triplet yield varies, with a highest yield at the lowest fluence. This is likely due to some bimolecular annihilation events between singlets or triplet pairs. The calculated yield at lower fluences exceeds 200%, which implies a systematic error in the calculation, likely due to using the solution absorption cross sections in the solid state. However, we note that these figures are in the correct order of magnitude for quantitative generation of the triplet pair state, as expected from the fact that singlet fission to the pair is ~100 times faster than the radiative rate, while in dilute (<1%)

films in PS the PLQE is ~97% indicating a lack of competitive non-radiative decay pathways for the singlet. This implies that the SF yield should be 198% with no annihilation.

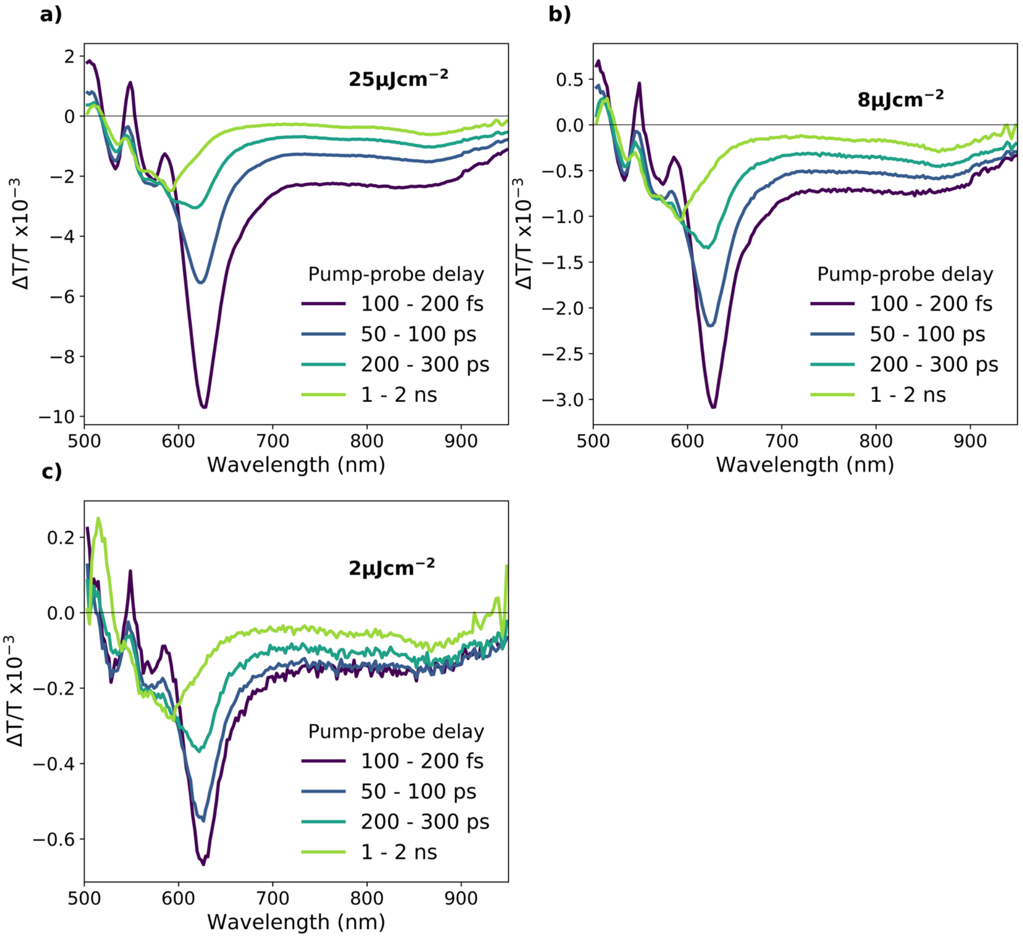

Supplementary Figure 17: Transient absorption spectra for 3 different excitation densities on a 7% TTBP in PS film. Excitation was performed with a 17 kHz 200 fs 550 nm pulse. The probe was a 34 kHz broadband probe generated in a YAG crystal, with the delay relative to the excitation pulse controlled mechanically.

Supplementary Note 5: Magnetic Field Effect on Photoluminescence

The Merrifield model used to fit the magnetic field effect (MFE) in Main Text Figure 2d is described in Supplementary Equation 5.1. When constrained to a steady state with constant populations of *S* and *P*$_i$, we obtain photoluminescence as a function of **B** shown in Supplementary Equation 5.2. The photoluminescence depends on α_i, the singlet projection of the pair states P_i, calculated according to the pair Hamiltonian. This Hamiltonian includes the exchange interaction with coupling parameter *J*, the intra-triplet zero-field splitting interaction parameterised by the zero-field splitting parameter *D*, and the Zeeman interaction with the external **B** field. The parameter a is the ratio of singlet fission rate to radiative rate and does not strongly affect the lineshape, but predominantly modulates the magnitude of the MFE (Supplementary Figure 19). The ratio ε, of the rate of triplet fusion (from pair-state back to singlet) to the rate of pair dissociation, does affect the line shape, with a higher ε required for a higher crossing point (Supplementary Figure 18).

$$\begin{array}{cccccccc} G & \searrow & & \gamma_+\alpha_i & & \gamma_d & \\ & & S & \rightleftharpoons & \{P_i\} & \rightarrow & T+T \\ & \gamma_r & \downarrow & \gamma_-\alpha_i & & & \end{array} \qquad 5.1$$

$$PL(B) = \gamma_r S(B) = G\left(1 + a\sum_i \frac{\alpha_i}{1+\varepsilon\alpha_i}\right)^{-1} \text{ where } G = \text{generation rate, } a = \frac{\gamma_+}{\gamma_r} \text{ and } \varepsilon = \frac{\gamma_-}{\gamma_d} \quad 5.2$$

In addition to the kinetic parameters, the MFE is dependent on the strength of the inter-triplet exchange coupling *J* within the pair states. The low field MFE effect only occurs when *J* is small relative to *D*, the dipolar interaction[4,5]. The exchange value depends on inter-triplet wavefunction overlap between molecules[6]. We note that strongly coupled pairs give little or no effect in the range of 0-200 mT that was explored in this study and so this experiment highlights only weakly coupled triplet pairs, without ruling out the presence of other, more strongly coupled pairs.

The fitting process was as follows: D and E, the zero field splitting parameters which define the dipolar interaction, were fixed at the value extracted from trESR simulations ($D = 1340$ MHz or 47.9 mT and $E = 28$ MHz or 1.0 mT). In order to fit to the lineshape measured in films of TTBP, we had to increase ε from around 1 in tetracene/TIPS-tetracene to 10, which brought the zero crossing point to the correct range (~150 mT). Supplementary Figure 18 shows how varying ε while keeping *J* low and a constant affects the MFE lineshape. a can also be varied, however this has a negligible effect on the lineshape and predominantly affects the magnitude of the effect. Supplementary Figure 19 shows how increasing a increases the magnitude of MFE. We set a to 200 from the rates obtained from transient absorption – the radiative rate is 22 ns (Supplementary Figure 14), while formation time of the pair state is of the order of 100 ps (Main Text Figure 2c), giving a ratio of around 200. Finally, we come to *J*, for which we found the best fit at 0.32D = 1.7 μeV. Supplementary Figure 20 shows the dramatic changes to lineshape resulting from small changes in *J*. At $J > 0.5D$ the dips from the level anticrossings at *J*, $3J/2$ and $3J$ are visible in the simulation. At $J > 3D$ the level anticrossings occur outside the range of fields investigated and there is negligible MFE in 0-200 mT.

While the MFE results show clearly that singlet fission takes place in TTBP and TTBP-PS blends, and that all of the photoluminescence features are from fission-involved excited states, we note that the model used to simulate the lineshape is overly simplistic. While we have fit to a single "average" exchange coupling strength and a singlet set of kinetic parameters, there is likely to be a distribution of both within any sample. This can be due to the presence of multiple structural polymorphs in the sample and differently geometrically-arranged pairs within a single polymorph, as seen in TIPS-tetracene[6] and DPH[4].

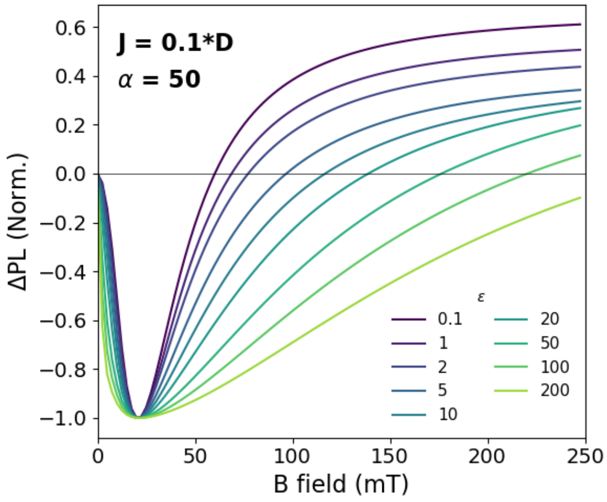

*Supplementary Figure 18: Example normalised MFEs for a range of epsilon and low J (0.1 D), showing how the lineshape, particularly the zero crossing point, change with epsilon. Calculated for at a single molecular orientation to the **B** field ($\theta = \pi/4$, $\varphi = \pi/2$).*

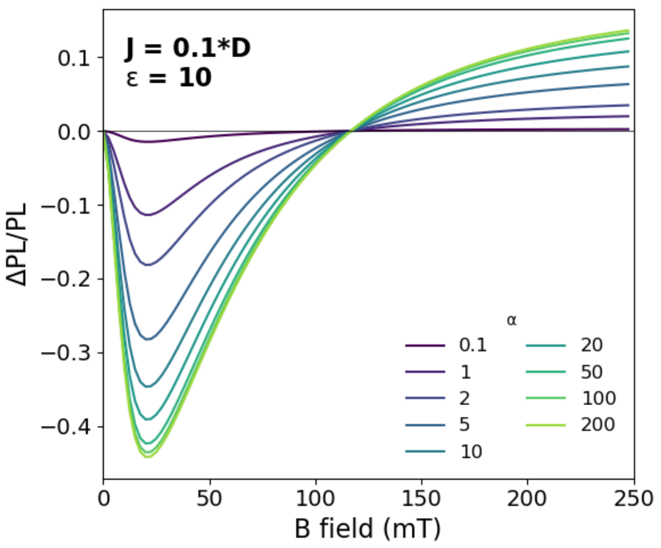

*Supplementary Figure 19: Example not-normalised MFEs for a range of alphas and low J (0.1 D), showing how the lineshape is unchanged with α, but the magnitude of the MFE increases with increasing α. Calculated for at a single molecular orientation to the **B** field ($\theta = \pi/4$, $\varphi = \pi/2$).*

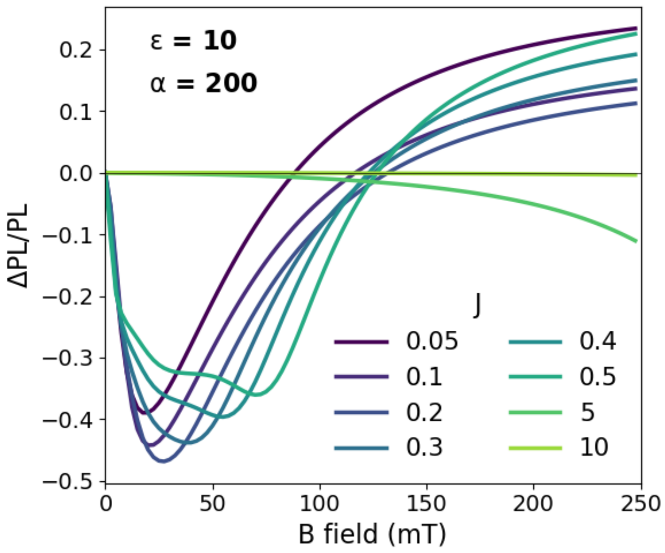

Supplementary Figure 20: Example normalised MFEs for a range of J showing how the lineshape, particularly the zero crossing point, change with J. Calculated for at a single molecular orientation to the B field ($\theta = \pi/4$, $\varphi = \pi/2$).. J values are in units of D.

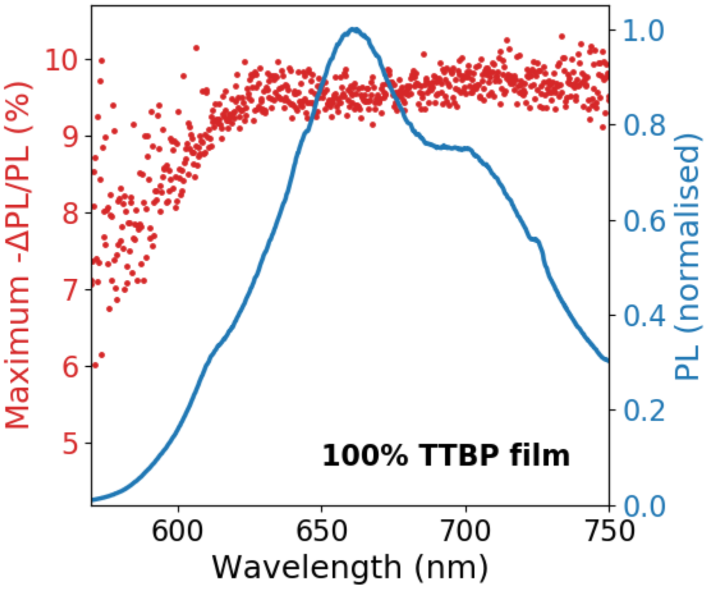

Supplementary Figure 21: Spectral dependence of the magnitude of the MFE in 100% TTBP film (red, dots) compared to the steady state PL spectrum at $\boldsymbol{B} = 0$ (blue line).

Supplementary Note 6: trESR

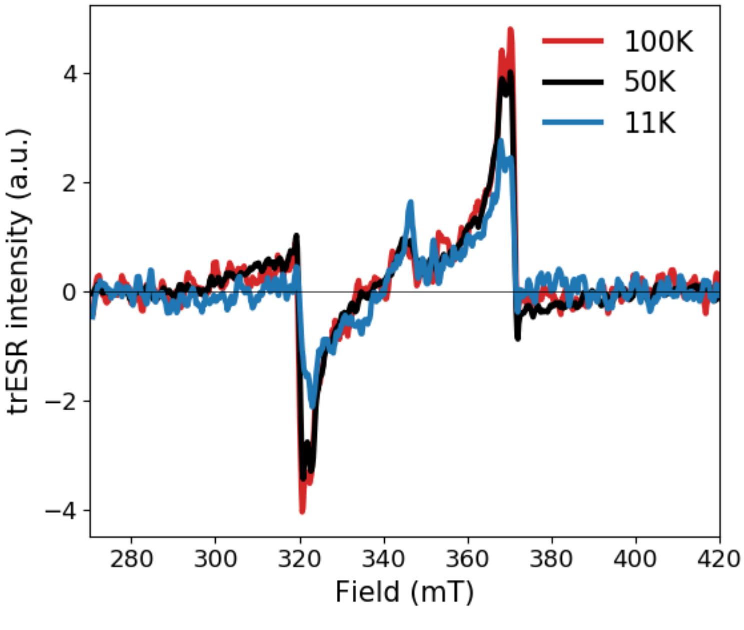

Supplementary Figure 22: Time resolved ESR spectra at 11 K (blue), 50 K (black) and 100 K (red), for a pure TTBP dropcast film, showing the higher population of free triplets at higher temperatures, due to temperature activated separation of the pair state. The film orientated at 0° to the $\boldsymbol{B_0}$ field. Signal is integrated from 0.3-1.5 μs after laser excitation.

The dependence of the trESR signal intensity on temperature, shown in Supplementary Figure 22, demonstrates a smaller population of free triplets at lower temperature. From 100 K to 11 K, the size of the signal halves. The difference in population is a factor of 2 or greater, as the resonator Q-factor increases at low temperatures, which leads to a larger signal for a given population of free triplets.

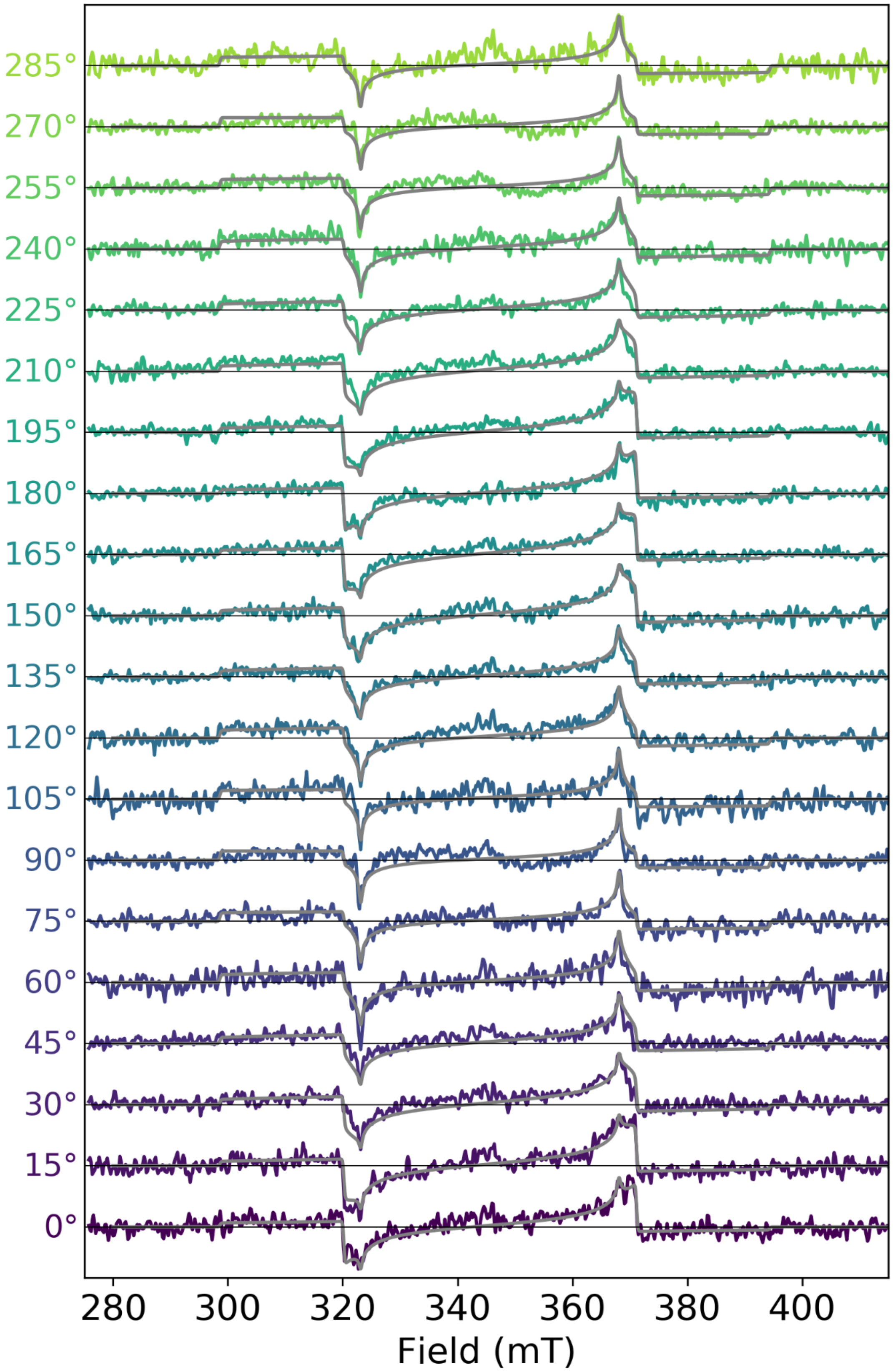

Supplementary Figure 23 Rotational dependence of trESR signal at 50 K integrated from 0.4-0.8 μs after laser flash, showing the preferential orientation of the polycrystalline domains to the substrate. Angles labelled are read out from the goniometer indicating angle of the substrate plane to the $\boldsymbol{B_0}$ *field. Simulations shown in grey using EasySpin*[7]*, detailed below.*

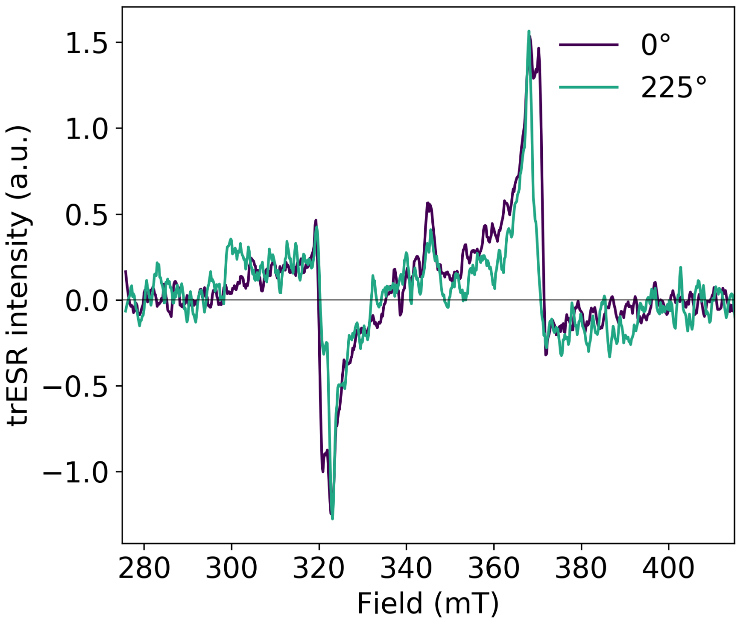

Supplementary Figure 24: Direct comparison of trESR spectra at 2 different sample orientations, 0° and 225°, at 50 K. Signal is integrated from 0.4-0.8 μs after laser excitation.

Supplementary Figure 23 and Supplementary Figure 24 show a clear dependence on the angle of the sample substrate relative to the magnetic field. Considering the fact that two crystal structures were reported for TTBP by Müller et al.[8], two scenarios could result in the observed orientation dependence. Firstly, the presence of the brick wall polymorph in polycrystalline domains with partial ordering due to some orientation selective crystallization relative to the substrate. Secondly, the presence of both polymorphs (brick wall and leaning double stack) also with the presence of partial ordering due to orientation selective crystallisation. The crystal structure of the leaning stack polymorph suggests that its presence alone would not lead to strong orientation dependent trESR spectra. While our partial ordering simulation does not explicitly account for multiple polymorphs, it does not rule them out.

We simulate the rotation dependent trESR spectra using EasySpin[7] and a custom model representing the partial ordering in the system, due to orientation selective crystallization relative to the substrate. The simulated spectra shown in Supplementary Figure 23 and Supplementary Figure 26 are based on the ZFS parameters being $D = 1340$ MHz (47.9 mT) and $E = 28$ MHz (1.0 mT), with $g = 2.000$ and a linewidth of 0.4 mT.

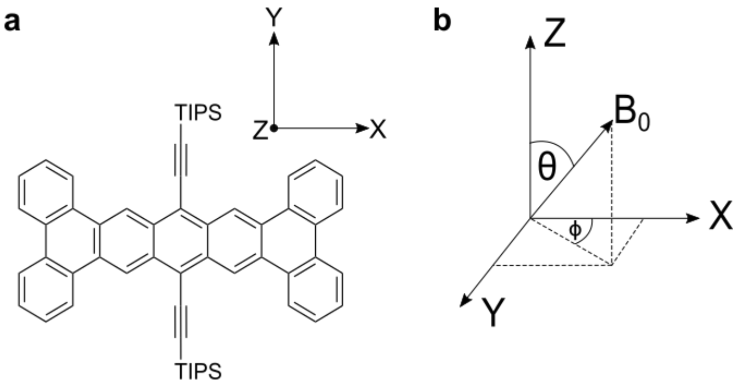

Supplementary Figure 25: Molecular axes of TTBP, with the x axis along the long axis of the molecule. b) shows the definitions of θ and φ which describe the angle between the ***B₀*** *direction and the molecular axes.*

The ZFS axes are assumed to be the same as for pentacene, where the molecular X axis is along the long axis of the conjugated core[9–11]. We expect the TTPB molecules to preferentially align this molecular X axis normal to the substrate plane. At a sample orientation of 0°, the substrate normal is perpendicular to the $\mathbf{B_0}$ direction and parallel to the incident laser beam. In this sample orientation the molecular X axis will also be preferentially aligned perpendicular to the $\mathbf{B_0}$ direction (parallel to the substrate normal). As defined by EasySpin and shown in Supplementary Figure 6.4b, θ is the angle between the molecular Z axis and the magnetic field vector, while φ is the angle between the molecular X axis and the projection of the magnetic field vector on the XY plane of the molecular frame.

Due to the preferential alignment, rotation of the sample in our setup (around the axis of the ESR tube) leads to rotation in both the molecular XZ and XY planes depending on the initial crystallisation orientation of each crystallite. Hence, we create a custom function for each sample orientation based on Gaussian curves which represent the distribution of the partially ordered system relative to the $\mathbf{B_0}$ direction. The distributions at different sample orientations are shown in Supplementary Figure 26, where panels b) and c) show the distributions used for sample rotation from angle e.g. 180° to 270° (blue to red curves). At each sample orientation, we generate a 2-dimensional distribution in θ and φ. Supplementary Figure 6.5b,c show 1-dimensional cuts of this distribution in the θ and φ directions, with cuts taken at the peak of the φ and θ distributions respectively.

First, considering distributions in θ shown in Supplementary Figure 26b, partial ordering in the system means that when the sample orientation is 180°, the distribution peaks at $\theta = 90°$ (dark blue line in Supplementary Figure 26b). This means there is a larger probability of having molecules with their molecular Z axis perpendicular to $\mathbf{B_0}$ and their molecular X axis parallel to $\mathbf{B_0}$. When the sample is rotated to 270°, the distribution then peaks at $\theta = 0°$. This means it is more probable for molecules to have Z parallel to $\mathbf{B_0}$ than X parallel to $\mathbf{B_0}$. This can be visualised by looking at the higher signal at the Z and Z' transitions (the outer peaks) in the dark red line (sample orientation 270°) compared to the dark blue line (sample orientation 180°) in Supplementary Figure 26a.

Similarly, when considering the distributions in φ shown in Supplementary Figure 26c, at a sample orientation of 180°, the distribution is peaked for molecules oriented at $\varphi = 90°$. There is a larger probability of having molecules with their molecular X axis perpendicular and their molecular Y axis parallel to $\mathbf{B_0}$. When the sample is rotated to 270°, the distribution peaks at $\varphi = 0°$, meaning there is a larger probability of molecules having their X axis parallel/antiparallel to the $\mathbf{B_0}$ direction and their Y axis perpendicular. This can be seen in the change in strength of the Y and Y' transitions in Supplementary Figure 26a – stronger at sample orientation of 180° (dark blue), where more molecules are aligned with Y axis parallel to $\mathbf{B_0}$.

The simulations are calculated using the pepper function in EasySpin[7] and our custom distribution is accounted for by using the Exp.Ordering command. The peak positions of the distributions in θ go from 90° to 0° and in φ from 90° to 0° in steps of 15°, representing rotation of the sample about 90° from e.g. 180° to 270°, see Supplementary Figure 26. The FWHM for the Gaussians used in the θ distribution is 90° and in φ distribution it is 65°. To simulate the full rotation study from the goniometer read angles of 180° to 270°, the simulations are repeated every 90°, i.e. the simulation for angles 0° to 90° and 180° to 270° are the same.

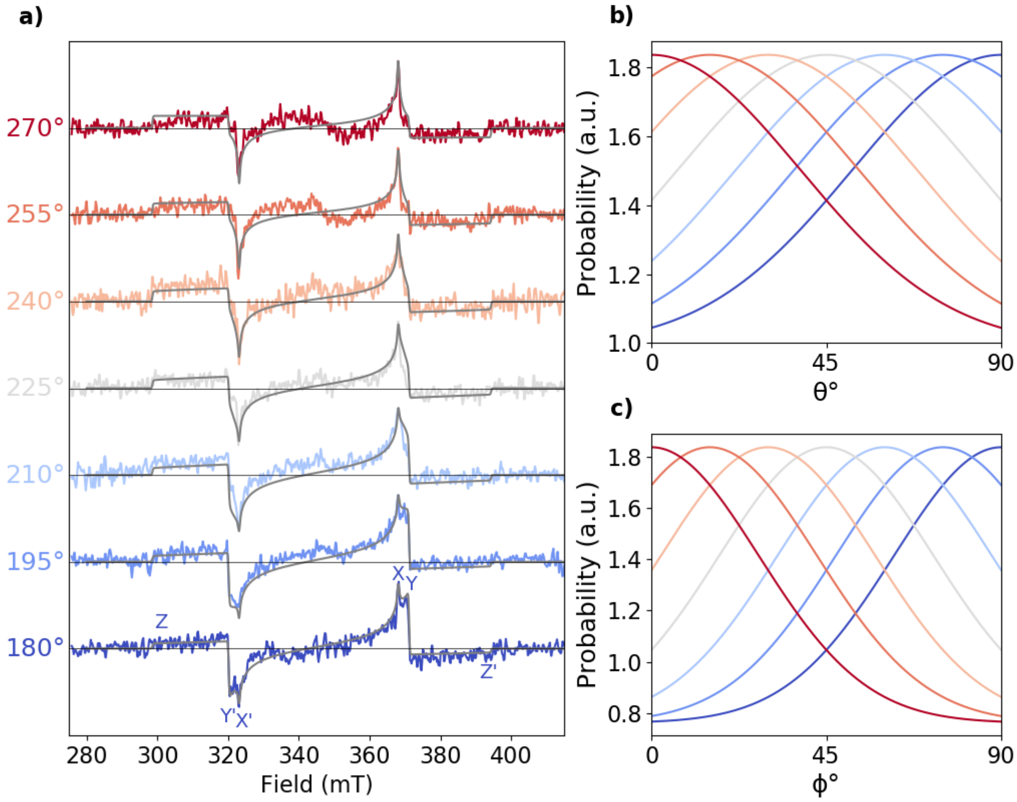

Supplementary Figure 26: a) trESR spectra from sample angle 180° to 270° with simulated spectra using EasySpin in grey. For the sample orientation of 180°, the transitions for different molecular orientations are labelled in the X, X' notation, where X/X' indicates the absorptive/emissive transition for molecules with X axis parallel to $\boldsymbol{B_0}$*. b) θ is the angle between the Z axis of the molecular frame and the magnetic field vector. The different curves represent the distribution in θ as the sample is rotated, each plotted at* $\varphi = \varphi_{max}$ *where the distribution is maximised. c) φ is the angle between X axis and the projection of the magnetic field vector on the XY plane of the molecular frame. The different curves represent the distribution in φ as the sample is rotated, each plotted at* $\theta = \theta max$ *where the distribution is maximised.*

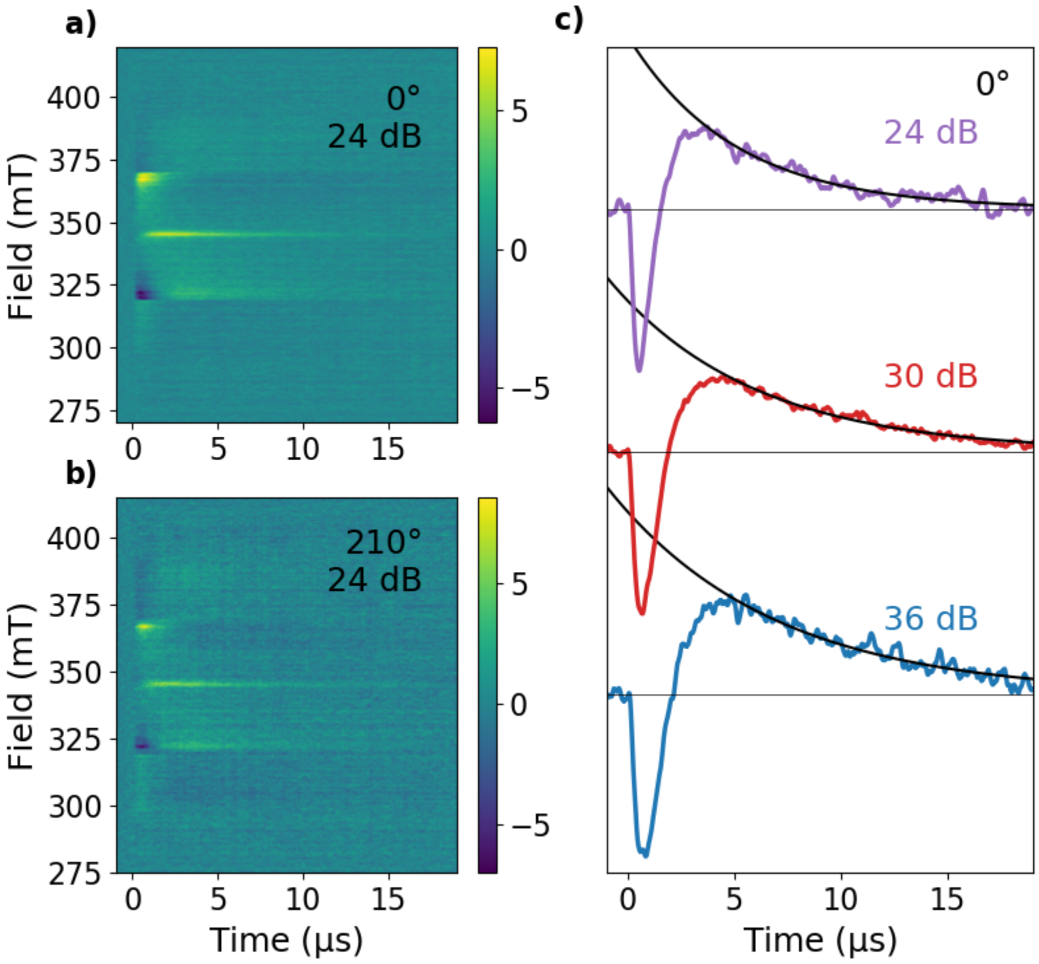

Supplementary Figure 27: a) Colormap showing evolution of trESR signal over time at 50 K with sample at 0° relative to the ***B₀*** *field and microwave attenuation of 24 dB b) Colormap showing evolution of trESR signal over time at 50 K with sample at 210° relative to the* ***B₀*** *field and microwave attenuation of 24 dB. c) Kinetics of the main emissive peak averaged over 321-323 mT in a) at 24 dB microwave attenuation compared to the same orientation and temperature with lower microwave powers of 30 dB and 36 dB. Black lines are exponential decays fitted to the transients after the inversion of the signal.*

The trESR signal decay times are all in the 5-8 μs range. Since the decay times do not change by more than a factor of 1.6 based on the fits going from 24 dB to 36 dB, we know the T_1 is at least 8 μs but cannot be much larger than ~10 μs. T_1 is an order of magnitude faster than the 100 μs ODMR component and therefore it is correct to assume that signal is from thermalized triplets.

Microwave attenuation (dB)	Decay time (μs)
24	5.0
30	6.8
36	7.7

Supplementary Table 3: Exponential decay times for different microwave attenuations, plotted in Supplementary Figure 27

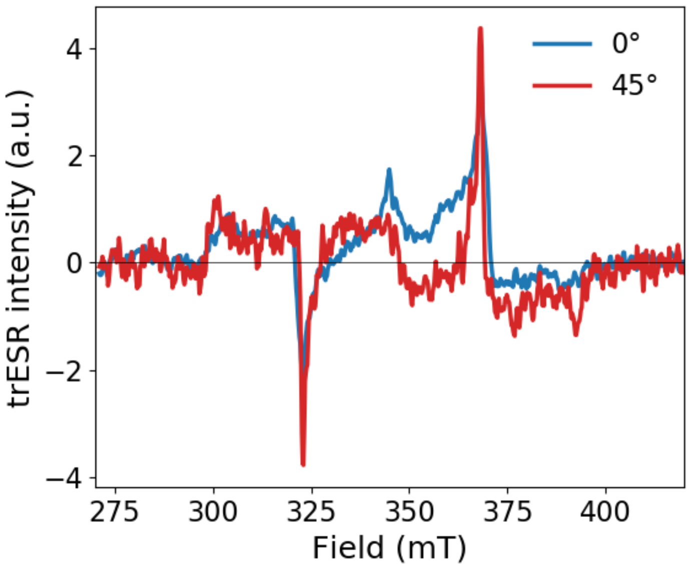

Supplementary Figure 28: trESR spectra for a sample of TTBP in polystyrene at 10% weight fraction, with the sample at angles 0° (blue) and 45° (red) at 50 K. Signal is integrated from 0.4-0.8 μs after laser excitation.

To investigate the degree of aggregation and singlet fission activity in the polystyrene blends, trESR spectra were recorded for a sample of 10% TTBP in polystyrene, of which two are shown in Supplementary Figure 28. The polarisation pattern of AEEAAE is identical to the pure dropcast TTBP sample, in Figure 3 of the main text, while the difference between 0° and 45° is very similar to that of 225° and 270° in Supplementary Figure 26a, indicating that there is still significant preferential alignment of the molecules relative to the substrate in the polystyrene blend.

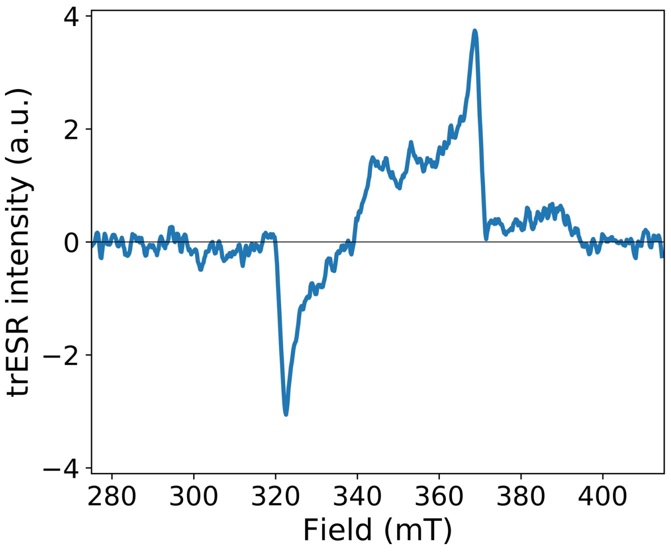

Supplementary Figure 29: trESR spectrum for a 1 mM frozen solution of TTBP in toluene at 50 K. Signal is integrated from 0.4-0.8 μs after laser excitation.

In contrast to all the solid samples of TTBP studied, the frozen dilute solution of TTBP has an trESR polarisation pattern of EEEAAA, compatible with intersystem crossing.

Supplementary Note 7: ODMR

ODMR was carried out with a different resonator to trESR, which required remounting the sample. Care was taken to orientate the sample at the same angle relative to the $\mathbf{B_0}$ field, and the ODMR measurements were intended to be taken with the sample at 0° to the $\mathbf{B_0}$ field. However, the shape of the ODMR spectrum, especially the sharp edge to the Z/Z' outer wings, indicates that the sample is likely to be closer to the orientation of 45° shown in Main Text Figure 3a.

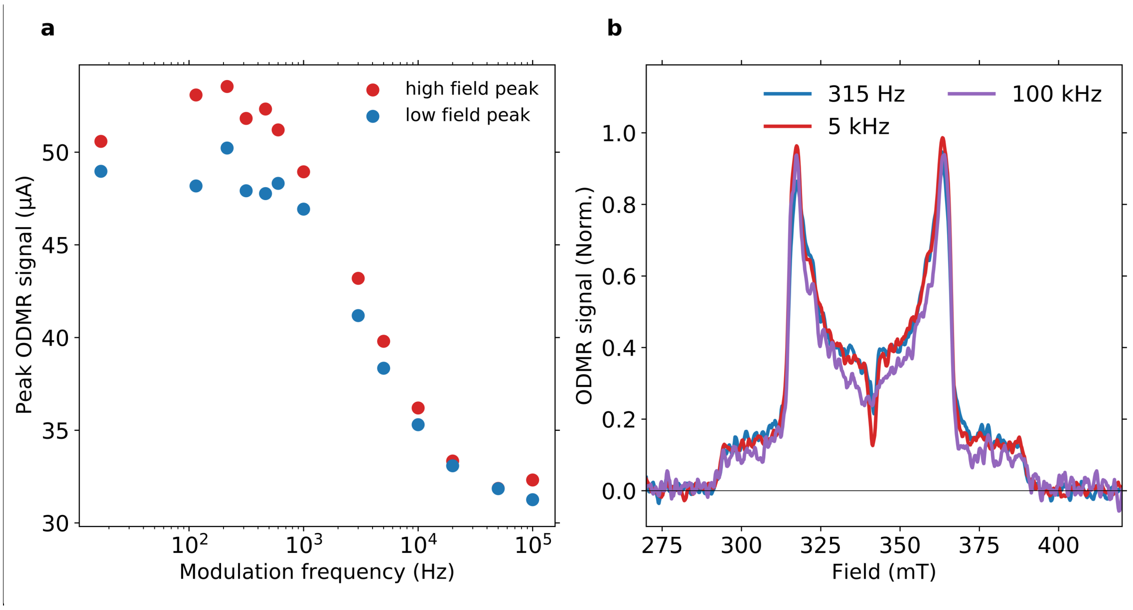

Supplementary Figure 30: a) Dependence of the intensity of low field (317.5 mT) and high field (363.5 mT) ODMR peaks on modulation frequency, at 36 mW CW 532 nm excitation. b) Comparison of ODMR spectra at different modulation frequencies, showing no change in polarisation of the signal at different timescales.

As the modulation frequency f_M was increased to 100 kHz, the limit of the lock-in amplifier, we observed a decrease in signal strength of around 40%. At 3 kHz, half of this 40% drop has occurred. Using the equation $\tau = \sqrt{3}/(2\pi f_M^*)$, we calculated a timescale of ~100 μs for 40% of the non-geminate TTA[12]. The remaining 60% occurs on a timescale faster than 3 μs. The 100 μs component takes place after the spin populations have depolarized (i.e. longer than the spin-lattice relaxation time T_1), meaning that this signal originates from a Boltzmann distribution of sublevel populations. However, the fast component, which occurs on a timescale faster than 3 μs, cannot be related to a Boltzmann distribution, as the trESR (Supplementary Figure 6.6) shows that there has not been enough time for the triplet sublevel populations to thermalize. Nevertheless, the ODMR spectrum and its polarization do not change with modulation frequency. The mechanism by which non-geminate TTA can generate this signal when not in a Boltzmann distribution is under further investigation.

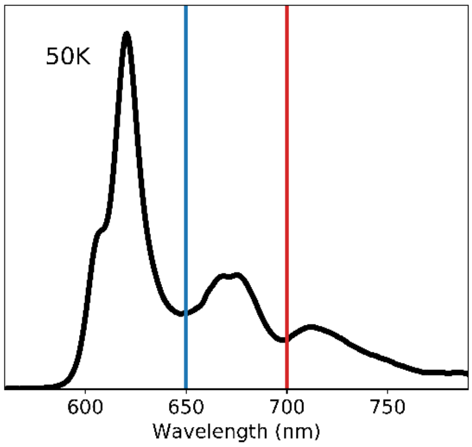

Supplementary Figure 31: 100% TTBP photoluminescence spectrum at 50 K with the positions of the short pass (blue line) and long pass (red line) filters shown.

Long pass and short pass filters allowed us to control the parts of the spectrum we were sensitive to. We were able to confirm that the lowest energy peak has a stronger contribution from non-geminate triplet-triplet annihilation than the high-energy peak. All parts of the spectrum have similar power and f_M dependence.

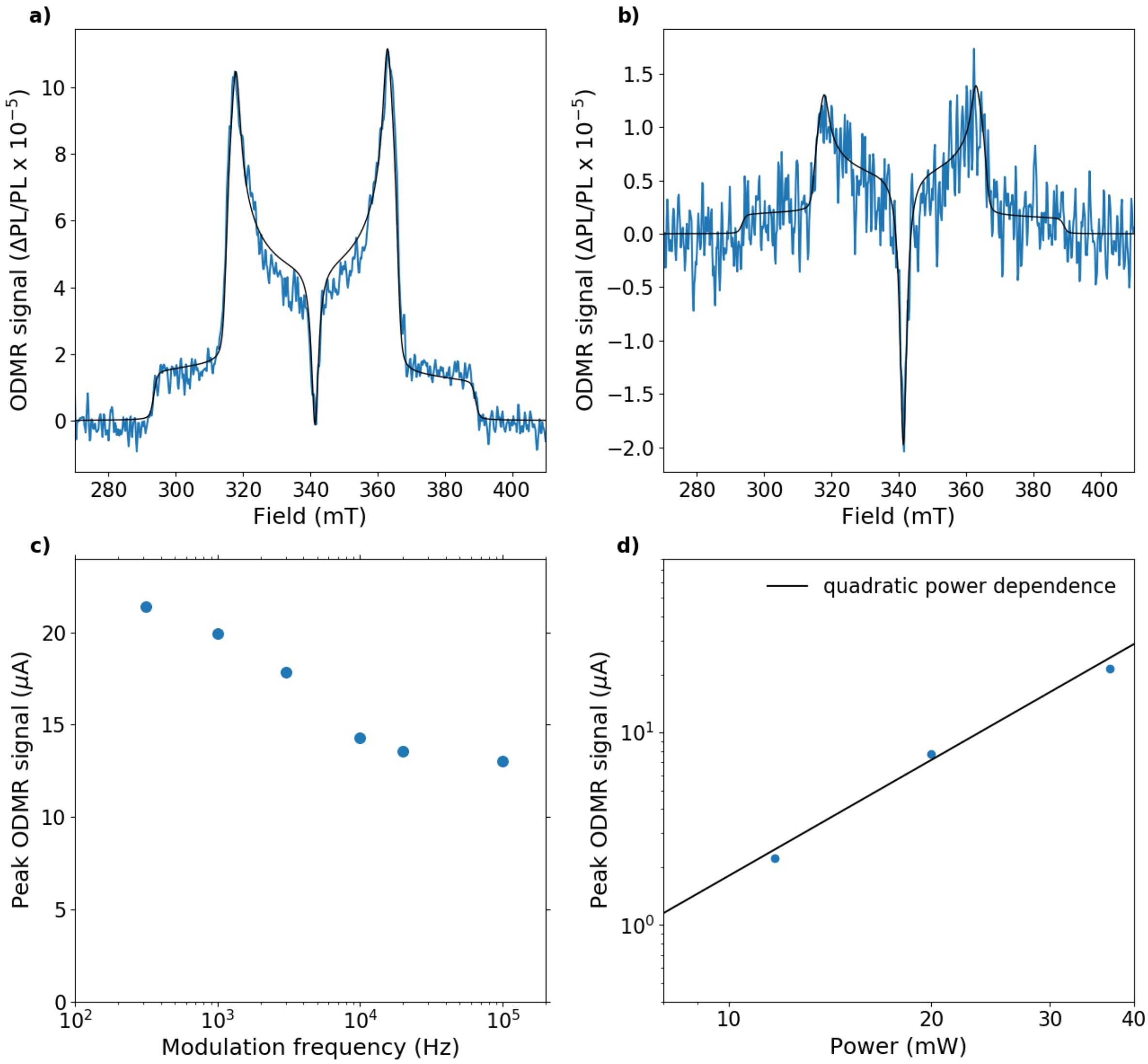

Supplementary Figure 32: ODMR using 650 nm short pass filter. a) ODMR signal at 36 mW CW 532 nm excitation, with simulation shown in black using EasySpin[7], with a thermal population at 50 K, the same D and E as for the trESR simulations ($D = 47.9$ mT, $E = 1.0$ mT) and linewidth = 1.3 mT. b) ODMR signal at 12 mW CW 532 nm excitation, with simulation shown in black. c) Dependence of peak ODMR signal on modulation frequency of the microwaves. d) Power dependence of the peak signal with a quadratic fit shown in black.

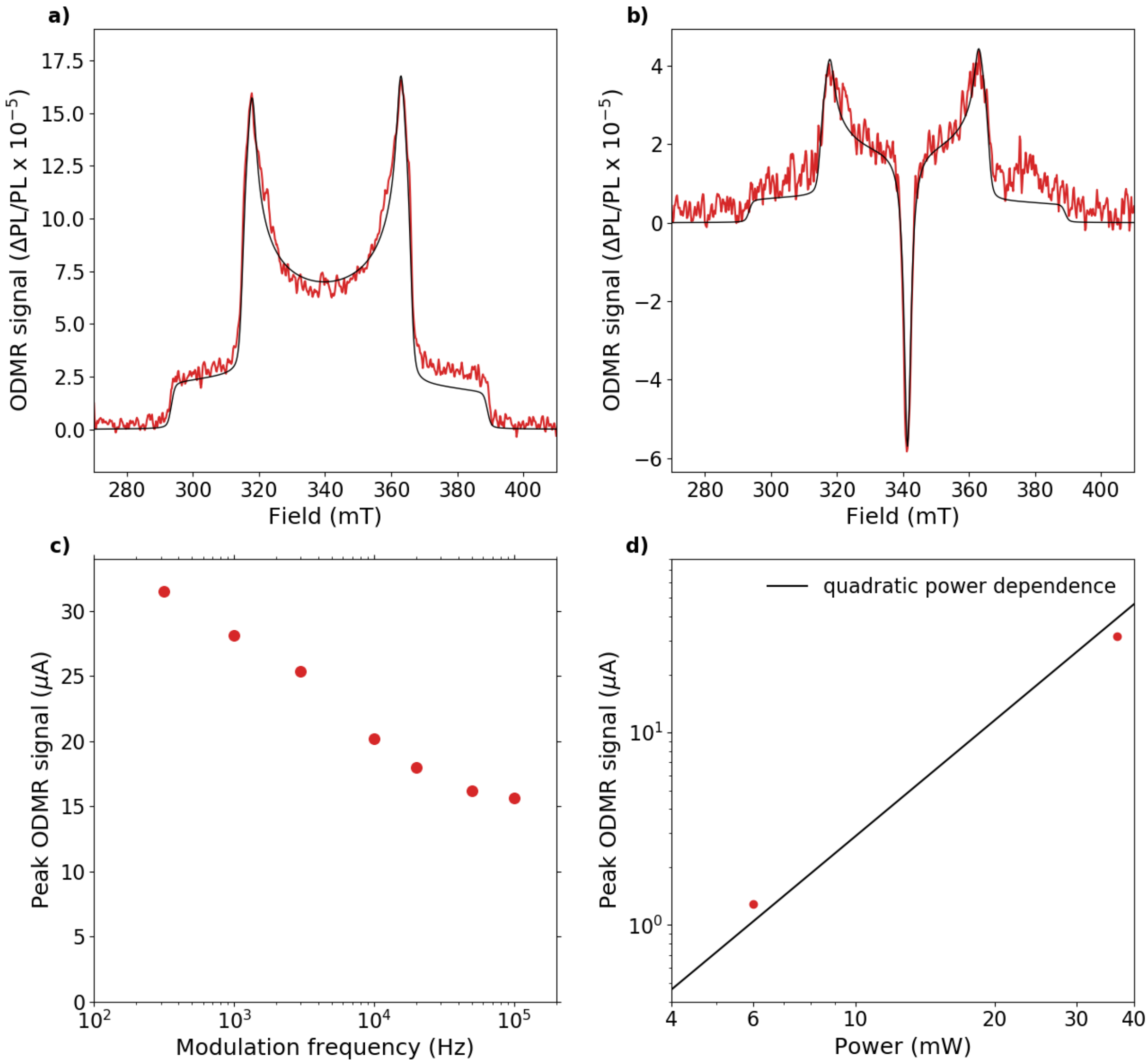

Supplementary Figure 33: ODMR using 700 nm long pass filter. a) ODMR signal at 36 mW CW 532 nm excitation, with simulation shown in black using EasySpin[7] with a thermal population at 50 K, the same D and E as for the trESR simulations ($D = 47.9$ mT, $E = 1.0$ mT) and linewidth = 1.3 mT. b) ODMR signal at 12 mW CW 532 nm excitation, with simulation shown in black. c) Dependence of peak ODMR signal on modulation frequency of the microwaves. d) Power dependence of the peak signal with a quadratic fit shown in black.

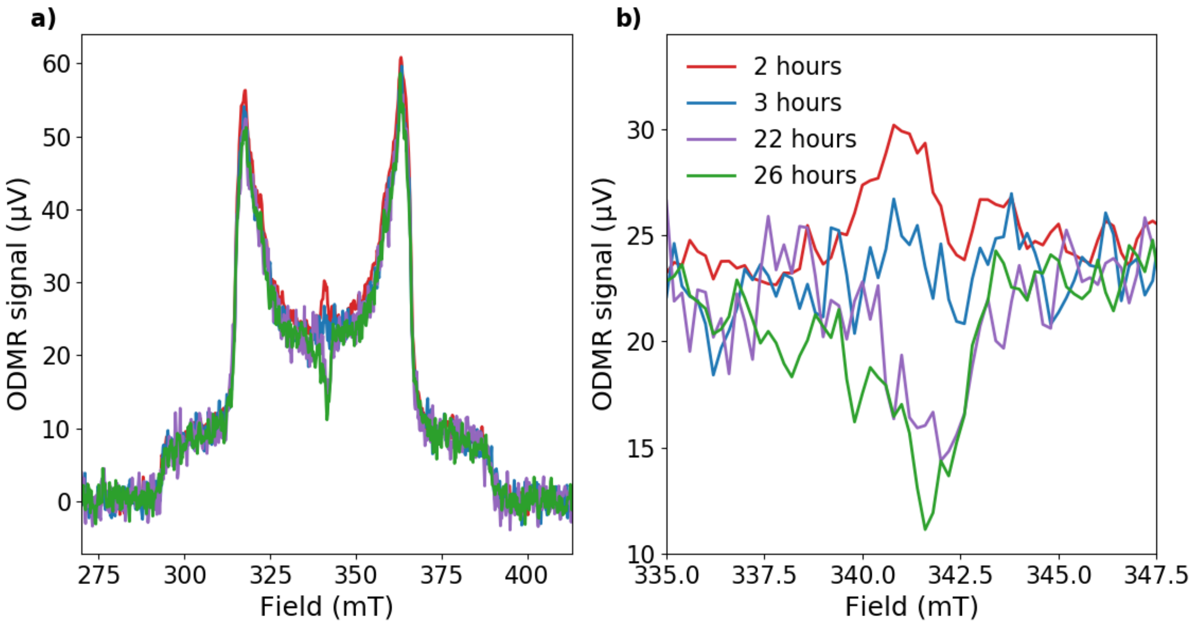

Supplementary Figure 34: Evolution of ODMR spectrum over time with 36 mW 532 nm CW excitation. Light-induced accumulation of persistent charges affects the central doublet peak.

The central doublet peak in the ODMR changed over time as the sample was irradiated with a 532 nm CW laser. Initially it was a positive peak, which quickly turned negative and then remained approximately constant. The power series data shown in Main Text Figure 5b was taken between the 22 hours purple spectrum and the 26 hours green spectrum, when there is only a modest increase in the magnitude of the doublet signal. The triplet peaks were unchanged throughout, so we attribute the doublet peak to a low density of light-induced, persistent charges which can undergo triplet-radical annihilation.

Supplementary References

1. Poletayev, A. D. *et al.* Triplet Dynamics in Pentacene Crystals: Applications to Fission-Sensitized Photovoltaics. *Adv. Mater.* **26**, 919–924 (2014).
2. Chow, P. C. Y., Albert-Seifried, S., Gélinas, S. & Friend, R. H. Nanosecond Intersystem Crossing Times in Fullerene Acceptors: Implications for Organic Photovoltaic Diodes. *Adv. Mater.* **26**, 4851–4854 (2014).
3. Stern, H. L. *et al.* Identification of a triplet pair intermediate in singlet exciton fission in solution. *Proc. Natl. Acad. Sci.* **112**, 7656–7661 (2015).
4. Wakasa, M. *et al.* What Can Be Learned from Magnetic Field Effects on Singlet Fission: Role of Exchange Interaction in Excited Triplet Pairs. *J. Phys. Chem. C* **119**, 25840–25844 (2015).
5. Bayliss, S. L. *et al.* Spin signatures of exchange-coupled triplet pairs formed by singlet fission. *Phys. Rev. B* **94**, 45204 (2016).
6. Bayliss, S. L. *et al.* Site-selective measurement of coupled spin pairs in an organic semiconductor. *Proc. Natl. Acad. Sci.* **115**, 5077–5082 (2018).

7. Stoll, S. & Schweiger, A. EasySpin, a comprehensive software package for spectral simulation and analysis in EPR. (2005). doi:10.1016/j.jmr.2005.08.013

8. Müller, M. *et al.* "Butterfly Wings" Stabilize Heptacene. *Chem. - A Eur. J.* **24**, 8087–8091 (2018).

9. Yang, T. C., Sloop, D. J., Weissman, S. I. & Lin, T. S. Zero-field magnetic resonance of the photo-excited triplet state of pentacene at room temperature. *J. Chem. Phys.* **113**, 11194–11201 (2000).

10. Sloop, D. J., Yu, H. L., Lin, T. S. & Weissman, S. I. Electron spin echoes of a photoexcited triplet: Pentacene inp-terphenyl crystals. *J. Chem. Phys.* **75**, 3746–3757 (1981).

11. Lubert-Perquel, D. *et al.* Identifying triplet pathways in dilute pentacene films. *Nat. Commun.* **9**, (2018).

12. Bayliss, S. L. *et al.* Geminate and Nongeminate Recombination of Triplet Excitons Formed by Singlet Fission. *Phys. Rev. Lett.* **112**, 238701 (2014).
